# Smoking‐Induced STC2^+^ Tumor Cells Drive Tumor‐Vascular Crosstalk in Laryngeal Squamous Cell Carcinoma via Spatial and Single‐Cell Transcriptomics

**DOI:** 10.1002/advs.202511932

**Published:** 2025-11-07

**Authors:** Yujie Shen, Lianchong Gao, Qiang Huang, Liang Zhou, Chengzhi Xu, Chunping Wu

**Affiliations:** ^1^ Department of Otolaryngology Shanghai Key Clinical Disciplines of otorhinolaryngology Eye Ear Nose & Throat Hospital Fudan University Shanghai 200025 P. R. China; ^2^ Shanghai Center for Systems Biomedicine Key Laboratory of Systems Biomedicine (Ministry of Education) Shanghai JiaoTong University Shanghai 200025 P. R. China; ^3^ Department of Otorhinolaryngology Kashi Prefecture Second People's Hospital Xinjiang Xinjiang Uygur Autonomous Region 844000 China

**Keywords:** laryngeal squamous cell carcinoma, smoking, tumor microenvironment, single‐cell RNA sequencing, spatial transcriptomics

## Abstract

Smoking‐associated laryngeal squamous cell carcinoma (LSCC) is characterized by high metastatic potential and poor prognosis. However, the underlying molecular mechanisms remain insufficiently understood. This study utilized single‐cell RNA sequencing (scRNA‐seq) and spatial transcriptomics to explore the heterogeneity of the tumor microenvironment in smoking‐associated LSCC. Thirteen distinct cellular subpopulations within the tumor microenvironment are identified, with STC2 and ITGA5 emerging as smoking‐associated prognostic markers. STC2 exhibited bifurcated differentiation within tumor epithelial cells, categorized as Tumor_C1 and Tumor_C2. The two subtypes are linked to vascular permeability and DNA replication pathways, respectively. Mechanistically, nicotine activated the JAK2/STAT3 signaling pathway through CHRNA5, resulting in direct STAT3 binding to the STC2 promoter and modulation of its transcription. STC2 subsequently upregulated TGFBI, which interacted with ITGA5 on endothelial cells, regulating vascular permeability and facilitating hematogenous dissemination of LSCC cells. Furthermore, STC2 knockdown altered F‐actin cytoskeletal dynamics by modulating small GTPase signaling, impairing filopodia formation and epithelial polarity restoration. This study elucidates the tumor–endothelial interactions mediated by STC2 and ITGA5 in smoking‐associated LSCC, emphasizing their roles in tumor progression and vascular permeability. These findings suggest potential prognostic biomarkers and therapeutic targets to improve the clinical management of smoking‐associated LSCC.

## Graphical abstract

This study identifies a smoking‐induced mechanism in LSCC whereby nicotine activates the CHRNA5–JAK2/STAT3 axis to drive STC2 transcription. STC2 upregulates TGFBI, which interacts with endothelial ITGA5 to regulate vascular permeability and promote metastasis. These findings highlight the STC2–TGFBI–ITGA5 axis as a critical mediator of tumor–endothelial crosstalk and a potential therapeutic target in smoking‐associated LSCC.

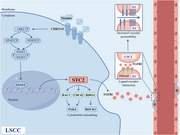

## 1 Introduction

Head and neck squamous cell carcinoma (HNSCC) arises from the mucosal epithelium of the larynx, oral cavity, nasal cavity, and pharynx,^[^ ^1^ ^]^ and is the sixth most common cancer worldwide.^[^ ^2^, ^3^ ^]^ Laryngeal squamous cell carcinoma (LSCC) represents over 95% of all malignant laryngeal tumors and is one of the most prevalent subtypes of HNSCC.^[^ ^4^ ^]^ Due to its insidious onset and nonspecific symptoms, LSCC is often diagnosed at advanced stages, contributing to a poor prognosis marked by high recurrence and metastasis rates.^[^ ^5^ ^]^ Despite advancements in treatment, improving long‐term survival and quality of life in head and neck oncology remains a significant challenge.

The pathogenesis of LSCC is multifactorial, involving tobacco use, alcohol consumption, nutritional deficiencies, immune dysregulation, and exposure to environmental carcinogens.^[^ ^1^ ^]^ Smoking is a well‐established risk factor for LSCC development and progression, with numerous studies linking it to increased incidence, recurrence, metastasis, and poor clinical outcomes.^[^ ^6^, ^7^, ^8^, ^9^ ^]^ These findings highlight the urgent need to explore the molecular mechanisms connecting tobacco exposure to LSCC progression, which may reveal novel biomarkers and therapeutic targets for high‐risk populations.

Nicotine, a primary constituent of both traditional cigarette smoke and electronic nicotine delivery systems, has attracted significant attention for its biological effects.^[^ ^10^ ^]^ However, the complex intercellular interactions within the nicotine‐modulated tumor microenvironment (TME) in LSCC remain poorly understood due to the inherent heterogeneity of these tumors. Stanniocalcin‐2 (STC2), implicated in invasion and poor prognosis in several cancers,^[^ ^11^ ^]^ has not been investigated for its regulation by smoking or its role in the LSCC‐specific TME crosstalk. Nicotine exerts its effects primarily through activation of nicotinic acetylcholine receptors (nAChRs).^[^ ^12^ ^]^ Our prior research demonstrated that nicotine activates the cholinergic receptor nicotinic alpha 5 subunit (CHRNA5), promoting interaction with RABL6 and triggering the JAK2/STAT3 signaling pathway to enhance LSCC metastasis.^[^ ^13^ ^]^ However, it remains unclear whether STAT3 directly regulates STC2 transcription in LSCC. Similarly, transforming growth factor beta‐induced protein (TGFBI), which interacts with integrins to promote tumor progression in other cancers,^[^ ^14^ ^]^ has not been explored for its interaction with endothelial integrin subunit alpha 5 (ITGA5) or its role in vascular permeability in LSCC.

This study integrated single‐cell RNA sequencing (scRNA‐seq) and spatial transcriptomics (ST) technologies to comprehensively characterize the cellular subpopulations and spatial interaction networks in smoking‐associated LSCC. Mechanistically, nicotine activates the CHRNA5/JAK2/STAT3 pathway to induce STC2, which promotes cytoskeletal remodeling through small GTPase signaling. ST analysis further reveals that STC2^+^ tumor cells interact with ITGA5^+^ endothelial cells (ECs) via TGFBI–ITGA5, thereby increasing vascular permeability. These findings uncover a novel mechanism by which smoking reshapes the TME at the single‐cell and spatial multi‐omics levels, providing a theoretical framework for therapeutic strategies targeting the STC2–TGFBI–ITGA5 axis.

## 2 Results

### 2.1 Single‐Cell Transcriptomic Landscape of Smoking Patients with Lscc

**Figure 1:**
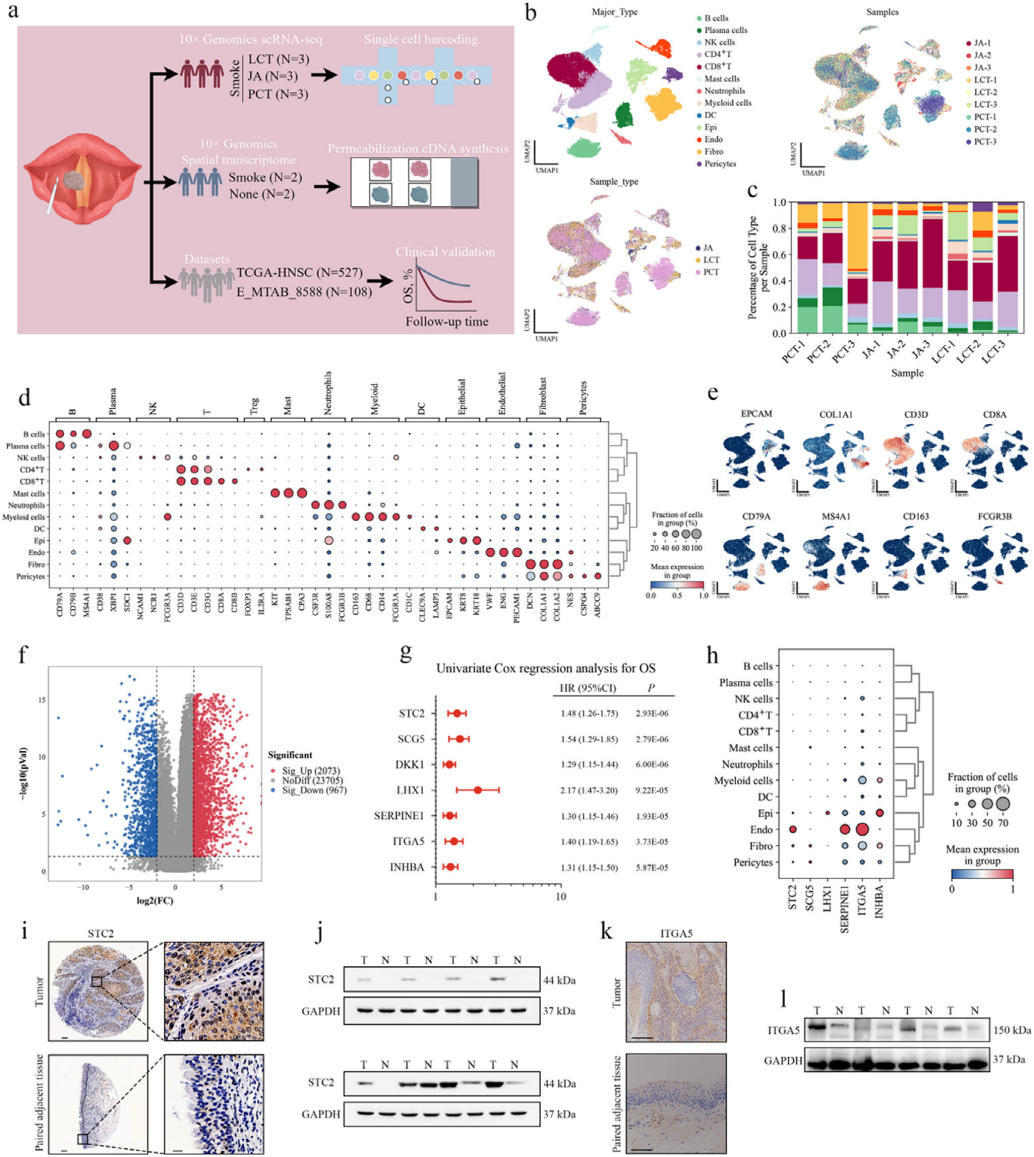
Single‐cell transcriptomic landscape of smoking patients with LSCC. a) Schematic overview of the experimental workflow (by Figdraw). Samples from smoking patients with LSCC were dissociated into single‐cell suspensions and subjected to scRNA‐seq using the 10× Genomics platform, without prior cell sorting. Tumor tissue sections were also analyzed using the 10× Genomics Visium method for ST analysis. b) UMAP visualizations of the single‐cell transcriptomic profiles from 44681 cells. Batch effects were corrected using the BBKNN algorithm in Python. c) Bar charts illustrating the proportions of the nine samples, stratified by donor and tissue source. d) Dot plots showing the average expression levels of selected canonical markers across identified cell clusters. Dot size reflects the proportion of cells expressing each marker within each cluster, with color indicating relative expression intensity. e) UMAP feature plots illustrating the expression patterns of selected canonical marker genes across 44681 unsorted cells. f) Volcano plot representing the mRNA expression profile in tumor versus normal tissues from smoking patients with HNSCC, analyzed using the R package “limma”. The screening criteria were: |log(fold change)| ≥ 2 and adj. *p* < 0.05. g) The top seven prognostic gene signatures ranked by statistical significance (*p*‐value) from the univariate Cox proportional hazards regression analysis. h) Dot plots displaying the average expression levels of prognostic gene signatures across identified cell clusters. Dot size reflects the proportion of cells expressing each marker within each cluster, with color indicating relative expression intensity. i) Representative IHC staining images of STC2 in tumor and matched adjacent normal tissues from a smoking patient with LSCC. Scale bars: 100 µm (left) and 20 µm (right). j) STC2 expression in tumor and matched adjacent normal tissues from smoking patients with LSCC. GAPDH served as a loading control. k) Representative IHC staining images of ITGA5 in tumor and matched adjacent normal tissues from a smoking patient with LSCC. Scale bar: 100 µm. l) ITGA5 expression in tumor and matched adjacent normal tissues from smoking patients with LSCC. GAPDH served as a loading control.

To analyze the cellular composition of LSCC, matched samples were surgically obtained from three anatomically distinct regions: proximal cancerous tissue, junction area, and lateral cancer‐adjacent tissue from three patients with smoking‐associated LSCC. These tissues were promptly processed for scRNA‐seq using the 10× Genomics platform, capturing transcriptomic data from the 3′‐end (**Figure** **1**a). Following stringent quality control to exclude damaged, apoptotic, or potential doublet cells, 44681 single‐cell transcriptomes were retained for further analysis (PCT, n = 18957; JA, n = 14520; LCT, n = 11204). Gene expression levels were normalized to adjust for sequencing depth and mitochondrial gene content. Principal component analysis (PCA) was performed based on highly variable genes across the dataset, followed by batch effect correction using the BBKNN algorithm to construct a batch‐balanced k‐nearest neighbor graph. A unified UMAP embedding was then generated based on the corrected neighbor graph, and Leiden clustering was applied to group the cells (Figure S1a, b, Supporting Information). While all thirteen major cell types were identified in the PCT, LCT, and JA samples from the three patients (Figure 1c), the infiltration levels of these cell types varied, reflecting the heterogeneity of LSCC progression.

Cells were classified into 13 major cell types based on canonical marker gene expression (Figure 1b–e; Figure S1c, Supporting Information), including CD8^+^ T cells (n = 13395) and CD4^+^ T cells (n = 9747), characterized by T‐cell receptor (TCR) signaling mediators CD3E and CD3G; natural killer (NK) cells (n = 1308), identified by NCAM1 and NCR1; fibroblasts (n = 5550), marked by COL1A1 and DCN; B cells (n = 4049), expressing MS4A1 and CD79B; plasma cells (n = 2275), with high expression of XBP1 and SDC1; epithelial cells (Epi; n = 2927), expressing EPCAM and KRT18; myeloid cells (n = 1639), identified by CD14 and CD68; endothelial cells (ECs; n = 1409), marked by PECAM1 and VWF; neutrophils (n = 522), expressing CSF3R and FCGR3B; dendritic cells (DCs; n = 513), characterized by CLEC9A and LAMP3; pericytes (n = 817), marked by CSPG4 and ABCC9; and mast cells (n = 530), identified by KIT and TPSAB1.

To identify key molecular targets, differential mRNA expression analysis was performed on RNA‐seq data from 413 smoking patients with HNSCC in the TCGA cohort using the R package “limma” (Figure 1f). The screening criteria were: |log(fold change)| ≥ 2 and adj. *p* < 0.05. Univariate Cox regression analysis was applied to these differentially expressed genes to identify prognostic gene signatures (Figure 1g). Annotation of the single‐cell RNA‐seq data revealed that these prognostic signatures were predominantly enriched in epithelial and EC populations (Figure 1h). Immunohistochemistry and Western blot analyses confirmed higher protein levels of STC2 and ITGA5 in tumor tissues from smoking patients with LSCC compared to paired adjacent normal tissues (Figure 1i–l).

### 2.2 Scrna‐Seq Reveals Stc2+ Epithelial Subpopulations Associated with Poor Prognosis and Aggressive Clinical Features in Smoking Lscc

**Figure 2:**
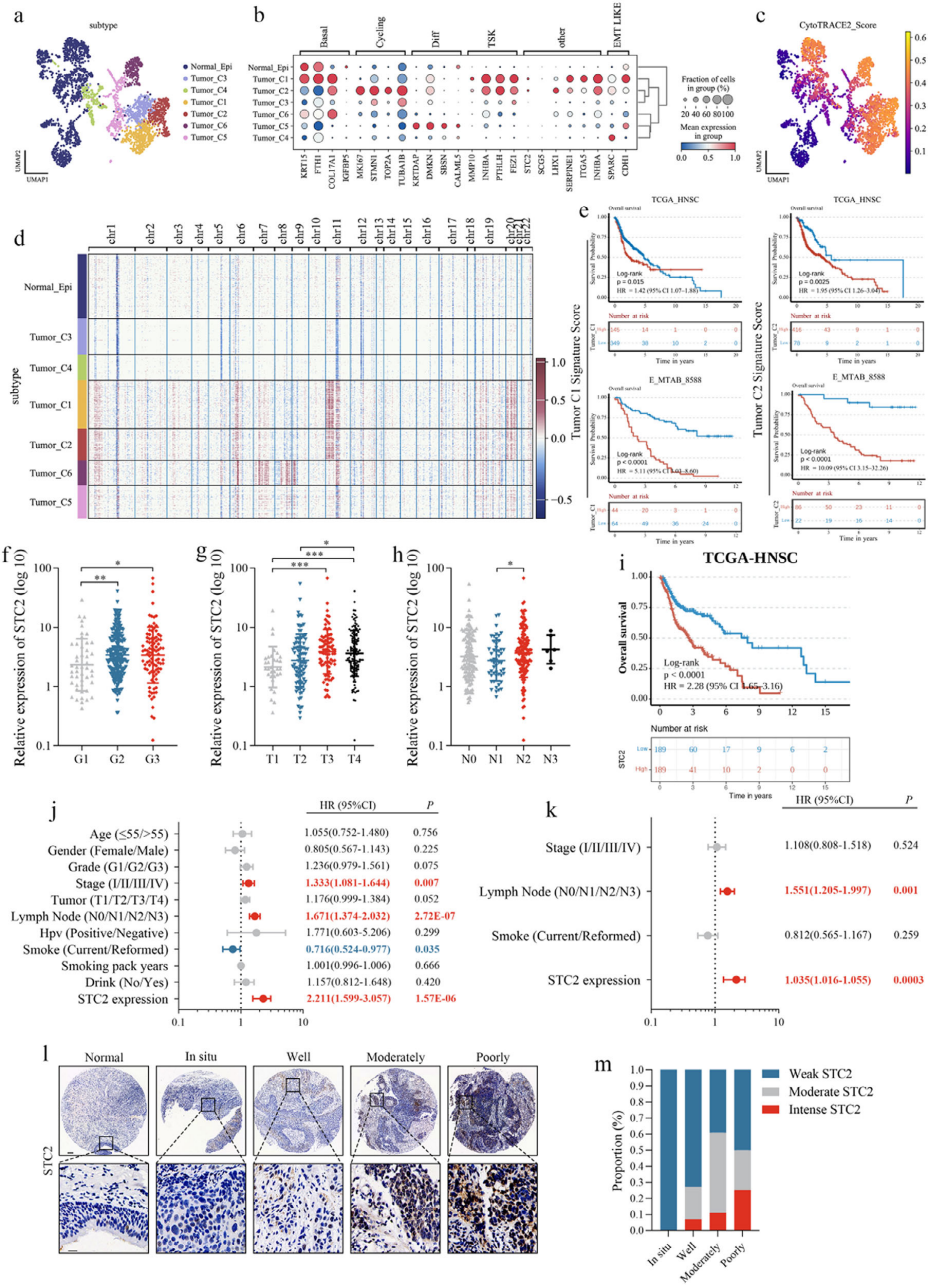
Identification of factors associated with poor prognosis and aggressive clinical features in smoking‐associated LSCC a) UMAP plot illustrating the classification of epithelial cells, with distinct colors representing different subtypes. b) Dot plot displaying the expression of selected genes across the seven epithelial cell subtypes. The color depth indicates the average expression value, and the size of each point reflects the percentage of cells expressing the gene. c) UMAP plots of epithelial cells, colored by CytoTRACE scores to represent cellular differentiation potential. d) Heatmap derived from InferCNVpy showing copy number alteration profiles across seven epithelial subpopulations. e) Kaplan–Meier survival curves of patients with HNSCC stratified by the signature scores of marker genes expressed in Tumor_C1 and Tumor_C2, based on the TCGA‐HNSC and E‐MTAB‐8588 cohorts. The optimal cutoff threshold was used for stratification, and *p*‐values were calculated using the Log‐rank test. Differential expression analysis of STC2 based on tumor grade f), T stage g), and N stage h). Statistical significance was determined using the Mann–Whitney U test, ^*^ *p* < 0.05, ^**^ *p* < 0.01, ^***^ *p* < 0.001. i) Kaplan–Meier curves comparing overall survival (OS) between patients with low and high STC2 expression, using the median expression value as the cutoff, with *p*‐values calculated using the Log‐rank test. Univariate j) and multivariate k) Cox regression analysis evaluating the independent prognostic value of STC2, variables with *p* < 0.05 in univariate analyses were entered into the multivariate model. l) Representative IHC staining images of STC2 in adjacent normal tissue, LSCC in situ, and well‐, moderately‐, and poorly differentiated LSCC tissues. Scale bar: 100 µm (top) and 20 µm (bottom). m) Proportions of different STC2 expression levels across LSCC in situ, and in well‐, moderately‐, and poorly differentiated LSCC tissues.

UMAP embedding of single‐cell transcriptomes revealed seven epithelial clusters, including one normal and six tumor‐derived subtypes (Tumor_C1–C6) (**Figure** **2**a). Tumor_C1 and Tumor_C2 showed upregulation of STC2, LHX1, INHBA, SERPINE1, and ITGA5 (Figure 2b). CytoTRACE analysis assigned the highest stemness scores to these two clusters (Figure 2c). InferCNVpy analysis identified focal amplifications on chromosomes 11 and 20, distinguishing Tumor_C1 and Tumor_C2 and accounting for their elevated CNV burdens (Figure 2d). Kaplan–Meier analyses (TCGA‐HNSC and E‐MTAB‐8588) demonstrated that the top marker genes of Tumor_C1 and Tumor_C2 (Table S5, Supporting Information) were associated with poor overall survival (OS) in LSCC (Figure 2e). Notably, STC2 was upregulated in both the highly proliferative Tumor_C2 cluster and the quiescent Tumor_C1 cluster. Pathway enrichment analysis indicated that Tumor_C1 was primarily enriched in extracellular matrix (ECM)‐associated pathways, suggesting its role in tumor–microenvironment interactions, while Tumor_C2 showed enrichment in cell cycle‐associated pathways, reflecting its proliferative phenotype (Figure S1d,e, Supporting Information).

Further clinical analysis revealed a positive correlation between STC2 expression and tumor grade (Figure 2f), T stage (Figure 2g), and N stage (Figure 2h). Kaplan–Meier survival analysis indicated that high STC2 expression was associated with poorer OS (Figure 2i). Univariate (Figure 2j) and multivariate (Figure 2k) Cox regression analyses confirmed STC2 as an independent risk factor. Immunohistochemistry demonstrated upregulation of STC2 in LSCC tumor tissues compared to adjacent normal tissues (Figure 2l), with its expression inversely correlated with tumor differentiation (Figure 2m).

### 2.3 Nicotine‐Induced Upregulation of Stc2 is Directly Regulated by the Transcription Factor Stat3

**Figure 3:**
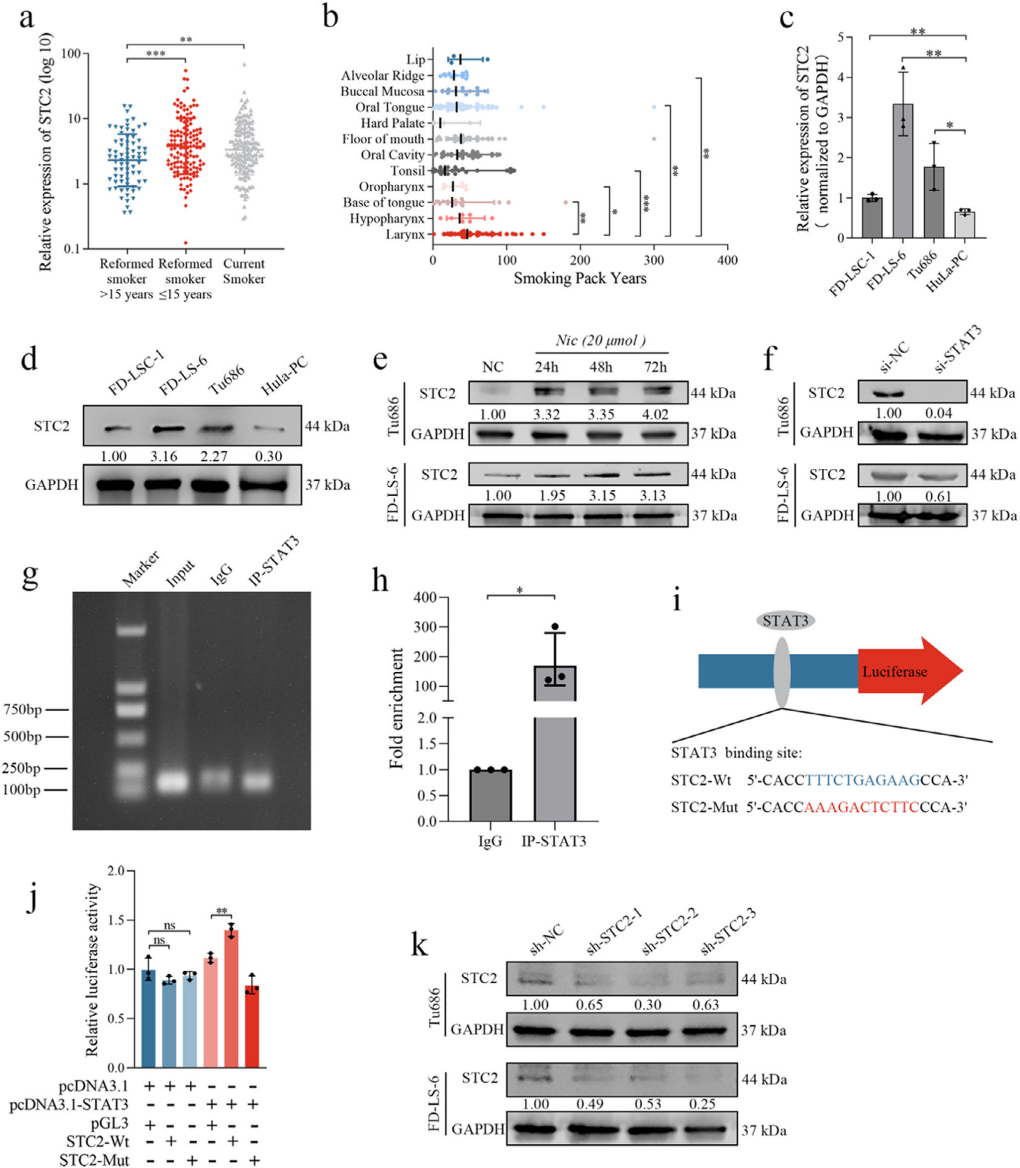
Nicotine promotes STC2 expression via transcription factor STAT3 in LSCC a) STC2 expression levels in tumors from patients with HNSCC categorized by smoking status. Statistical significance was determined using the Mann–Whitney U test, ^**^ *p* < 0.01, ^***^ *p* < 0.001. b) Comparison of smoking pack‐years among different HNSCC subtypes. Statistical significance was determined using the Mann–Whitney U test, ^*^ *p* < 0.05, ^**^ *p* < 0.01, ^***^ *p* < 0.001. c) Comparison of STC2 mRNA expression between normal laryngeal epithelial cells and LSCC cells. Data are presented as mean ± SD (n = 3). Statistical significance was determined using the unpaired *t*‐test, ^*^ *p* < 0.05, ^**^ *p* < 0.01. d) Comparison of STC2 protein expression between normal laryngeal epithelial cells and LSCC cells. GAPDH served as a loading control. e) STC2 protein expression in LSCC cells under varying durations of 20 micromolar nicotine stimulation. GAPDH served as a loading control. f) STC2 protein expression in LSCC cells following STAT3 knockdown. GAPDH served as a loading control. g) ChIP analysis of interactions between STAT3 and the STC2 promoter in HEK293T cells. h) ChIP‐qPCR assays detected the enrichment of DNA fragments in samples immunoprecipitated with the STAT3 antibody. Data are presented as mean ± SD (n = 3). Statistical significance was determined using the unpaired *t*‐test, ^*^ *p* < 0.05. i) Prediction of STAT3 binding sites with the STC2 promoter. j) Confirmation of the binding between STAT3 and the STC2 promoter using a dual‐luciferase reporter assay. Data are presented as mean ± SD (n = 3). Statistical significance was determined using the unpaired *t*‐test, ^**^ *p* < 0.01. k) Confirmation of STC2 protein expression in LSCC cells infected with sh‐NC, sh‐STC2‐1, sh‐STC2‐2, and sh‐STC2‐3 lentiviruses. GAPDH served as a loading control.

Clinical correlation analysis revealed a significant upregulation of STC2 expression in tumor tissues from patients with HNSCC who were current smokers (*p* = 0.0057) or had quit smoking within the past 15 years (*p* = 0.0009), compared to those who had abstained from smoking for more than 15 years (**Figure** **3**a). Additionally, cumulative tobacco exposure, quantified as smoking pack‐years (years smoked × packs per day), was higher in patients with LSCC than in those with other HNSCC subtypes (Figure 3b). These findings suggest a strong association between smoking and increased STC2 expression in HNSCC tumors, with particularly pronounced tobacco exposure in patients with LSCC.

qRT‐PCR (Figure 3c) and Western blot (Figure 3d) analyses confirmed significant upregulation of STC2 mRNA and protein levels in laryngeal carcinoma cell lines, FD‐LSC‐1 (*p* = 0.0066), FD‐LS‐6 (*p* = 0.0043), and Tu686 (*p* = 0.0302), relative to normal laryngeal epithelial cells (HuLa‐PC). Notably, the Tu686 and FD‐LS‐6 LSCC cell lines exhibited especially high STC2 expression. Based on previous studies,^[^ ^13^, ^15^ ^]^ LSCC cells were treated with 20 micromolar nicotine, resulting in a time‐dependent increase in STC2 expression following nicotine exposure (Figure 3e). Nicotine primarily acts through the nicotinic acetylcholine receptor α5 subunit (CHRNA5) to activate the JAK2/STAT3 signaling pathway, enhancing the metastatic potential of LSCC cells.^[^ ^13^ ^]^ Consistent with this, STAT3 knockdown via siRNA reduced STC2 expression in LSCC cells (Figure 3f), indicating that nicotine upregulates STC2 via a STAT3‐dependent mechanism.

To examine whether STAT3 directly regulates STC2, we performed ChIP assays in HEK293T cells, which confirmed the binding of STAT3 to the STC2 promoter (Figure 3g,h). We also conducted luciferase reporter assays by co‐transfecting STAT3‐pcDNA3.1 with either STC2‐wt‐pGL3 or STC2‐mut‐pGL3 constructs into HEK293T cells (Figure 3i). The results demonstrated that STAT3 enhanced the luciferase activity driven by the wild‐type STC2 promoter (*p* = 0.0038, Figure 3j), whereas no effect was observed with the mutant promoter. These findings provide evidence that STAT3 binds to the STC2 promoter and transcriptionally upregulates its expression. To assess STC2 function, Tu686 and FD‐LS‐6 cells were infected with sh‐NC and sh‐STC2 lentiviruses. Western blot validation confirmed the selection of Tu686 cells infected with the sh‐STC2‐2 lentivirus and FD‐LS‐6 cells infected with the sh‐STC2‐3 lentivirus for subsequent experiments (Figure 3k).

### 2.4 STC2 Knockdown Impairs Progression and Induces Epithelial‐Like Morphological Changes in Lscc Cells

**Figure 4:**
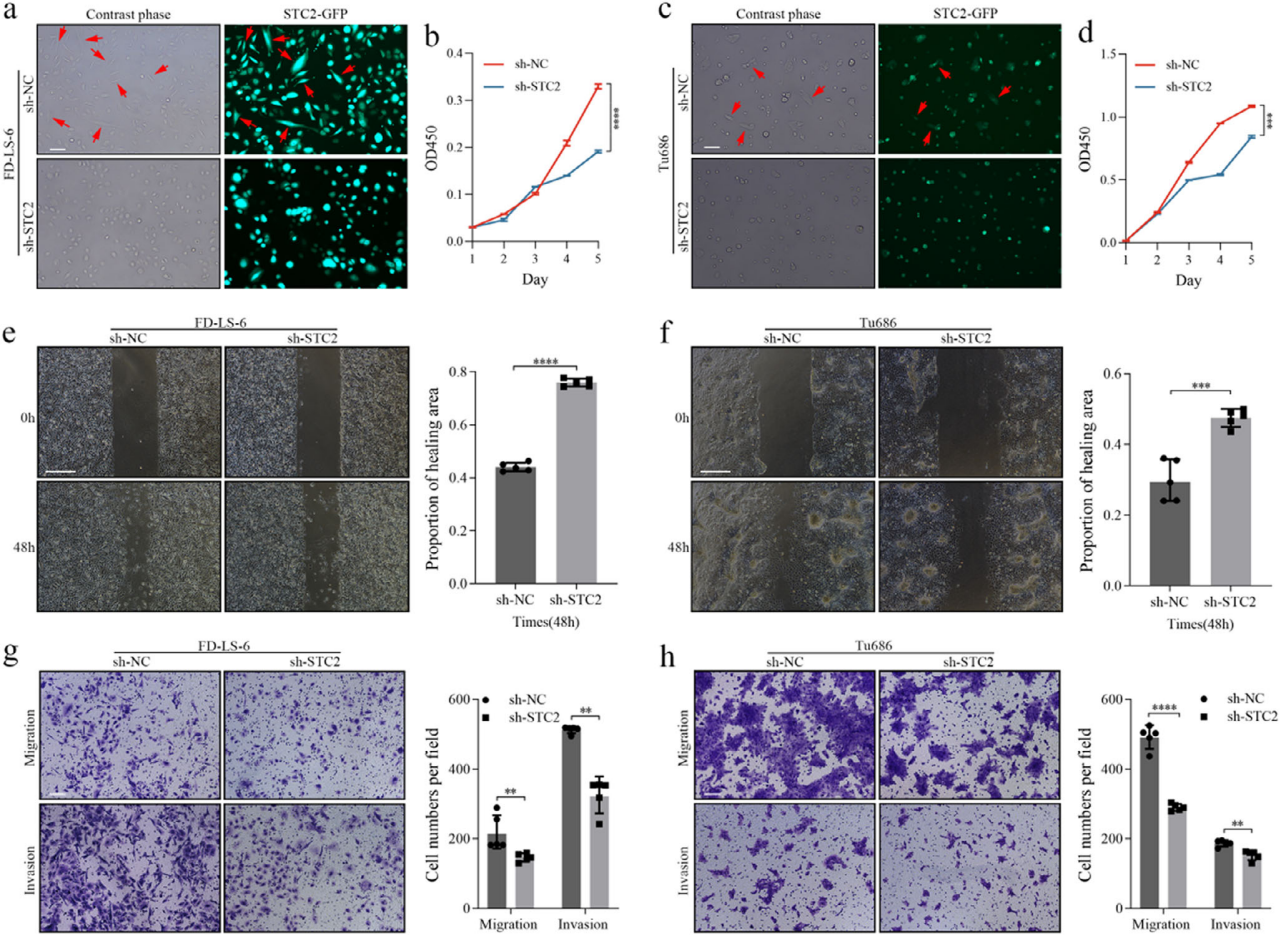
STC2 knockdown suppresses the progression of LSCC cells in vitro GFP‐labeled FD‐LS‐6 (a) and Tu686 (c) cells infected with sh‐STC2 and sh‐NC. Scale bar: 100 µm. Proliferative capacity of FD‐LS‐6 (b) and Tu686 (d) cells after STC2 knockdown as assessed by CCK‐8 assay. Data are presented as mean ± SD (n = 3). Statistical significance was determined using an unpaired *t*‐test, ^***^ *p* < 0.001, ^****^ *p* < 0.0001. Assessment of FD‐LS‐6 (e) and Tu686 (f) cell migration following STC2 knockdown via wound healing assays. Scale bar: 500 µm. Data are presented as mean ± SD (n = 5). Statistical significance was determined using an unpaired *t*‐test, ^***^ *p* < 0.001, ^****^ *p* < 0.0001. g) Migration and invasion of FD‐LS‐6 cells following STC2 knockdown assessed by transwell assays. Scale bar: 100 µm. Data are presented as mean ± SD (n = 5). Statistical significance was determined using Mann‐Whitney U test, ^**^ *p* < 0.01. h) Migration and invasion of Tu686 cells following STC2 knockdown assessed by transwell assays. Scale bar: 100 µm. Data are presented as mean ± SD (n = 5). Statistical significance was determined using an unpaired *t*‐test, ^**^ *p* < 0.01, ^****^ *p* < 0.0001.

GFP fluorescence analysis was conducted to evaluate the infection efficiency of sh‐NC and sh‐STC2 lentiviruses (**Figure** **4**a,c). Notably, STC2 knockdown induced an epithelial‐like morphological shift in LSCC cells, characterized by the restoration of epithelial polarity (Figure 4a,c). Functional assays corroborated these findings: CCK‐8 assays demonstrated a significant reduction in the proliferative capacity of FD‐LS‐6 (*p* < 0.0001) and Tu686 (*p* = 0.0003) cells following STC2 knockdown (Figure 4b,d). Wound healing assays revealed a substantial decrease in the migratory ability of LSCC cells post‐STC2 knockdown (Figure 4e,f), and transwell assays indicated a marked reduction in migration and invasion capacity in the STC2 knockdown group (Figure 4g,h).

### 2.5 Stc2 Knockdown Affected F‐Actin Cytoskeletal Remodeling by Regulating Small Gtpase Signaling

**Figure 5:**
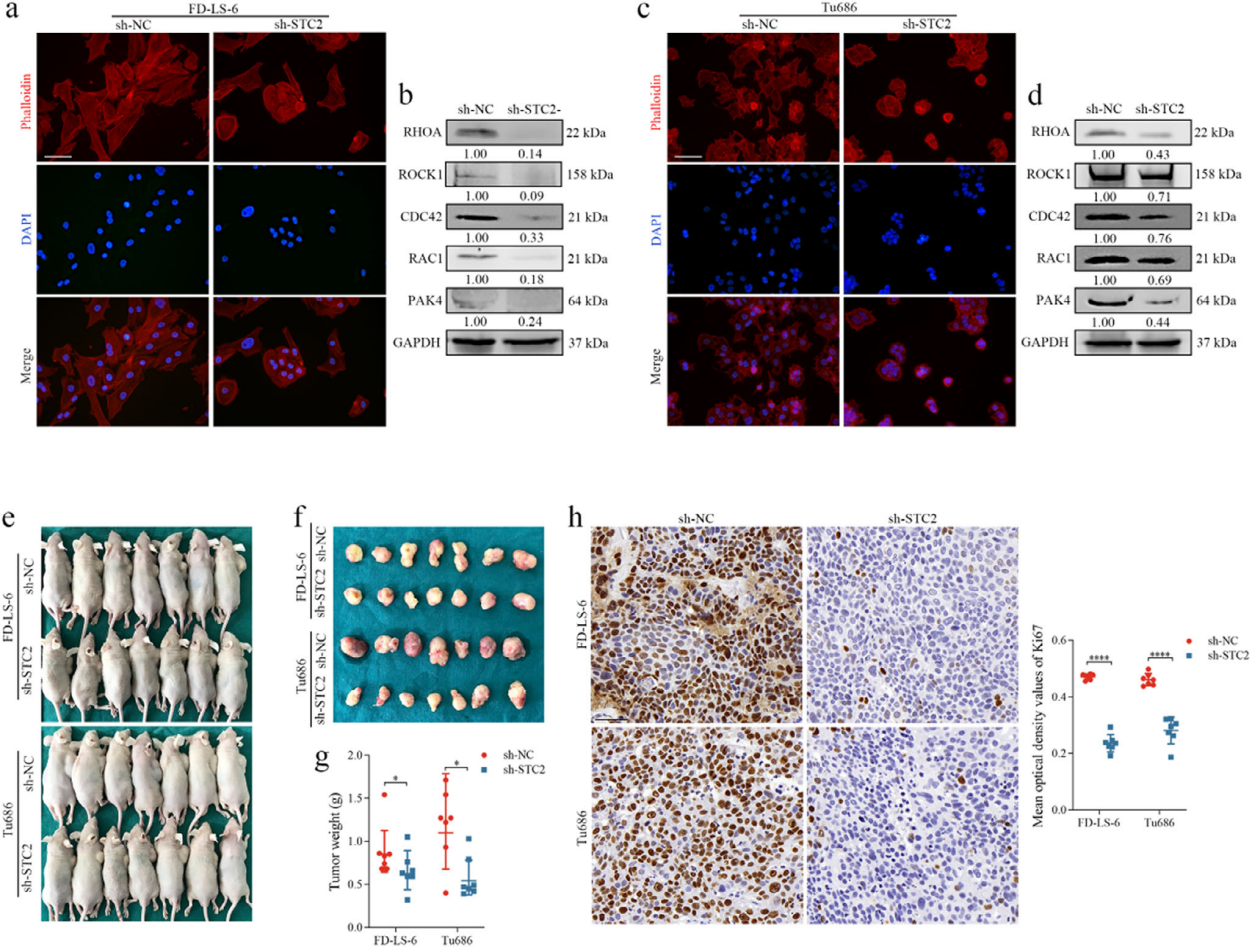
STC2 knockdown suppresses the malignant phenotype of LSCC cells by modulating F‐actin cytoskeletal remodeling. Representative fluorescence micrographs of FD‐LS‐6 (a) and Tu686 (c) cells stained with phalloidin (red) for F‐actin and DAPI (blue) for nuclei. Scale bar: 50 µm. Expression of small GTPases and their downstream effectors in FD‐LS‐6 (b) and Tu686 (d) cells following STC2 knockdown. GAPDH served as a loading control. e) Establishment of the subcutaneous xenograft model. f) Representative images of harvested xenograft tumors. g) Quantification of xenograft tumor weights across different groups. Data are presented as mean ± SD (n = 7). Statistical significance was determined using the Mann–Whitney U test, ^*^ *p* < 0.05. h) Representative IHC staining images of Ki67 in tumor tissues from different groups. Scale bar: 50 µm. Data are presented as mean ± SD (n = 7). Statistical significance was determined using an unpaired *t*‐test, ^****^ *p* < 0.0001.

To investigate the pro‐metastatic mechanisms of STC2 in LSCC cells, DAPI staining was used to visualize nuclei (blue), and phalloidin staining to label F‐actin (red) (**Figure** **5**a,c). STC2 knockdown induced an epithelial‐like morphological transition in LSCC cells, marked by the restoration of epithelial polarity and a significant reduction in filopodia and lamellipodia, structures essential for cell motility and migration. Western blot analysis further revealed that STC2 knockdown downregulated the expression of small GTPases RHOA, CDC42, and RAC1, along with their downstream effectors PAK4 and ROCK1 (Figure 5b,d). These findings suggest that STC2 knockdown suppresses small GTPase signaling, promotes F‐actin cytoskeletal remodeling, and reverses the metastatic phenotype of LSCC cells.

To assess the effects of STC2 knockdown in vivo, a subcutaneous xenograft model was established in mice (n = 7 per group, Figure 5e). Consistent with the in vitro results, tumor weight was significantly reduced in the FD‐LS‐6 (*p* = 0.0478) and Tu686 (*p* = 0.0245) groups following STC2 knockdown (Figure 5f,g). Additionally, Ki67 expression was markedly lower in the xenograft tissues of the sh‐STC2 group compared to the sh‐NC group (*p* < 0.0001, Figure 5h), indicating a decrease in proliferative capacity. This observation aligns with the highly proliferative phenotype of the STC2^+^ Tumor_C2 cluster identified in the single‐cell analysis.

### 2.6 scRNA‐Seq Reveals Itga5+ Ecs Associated with Poor Prognosis and Endothelial Cell Permeability

**Figure 6:**
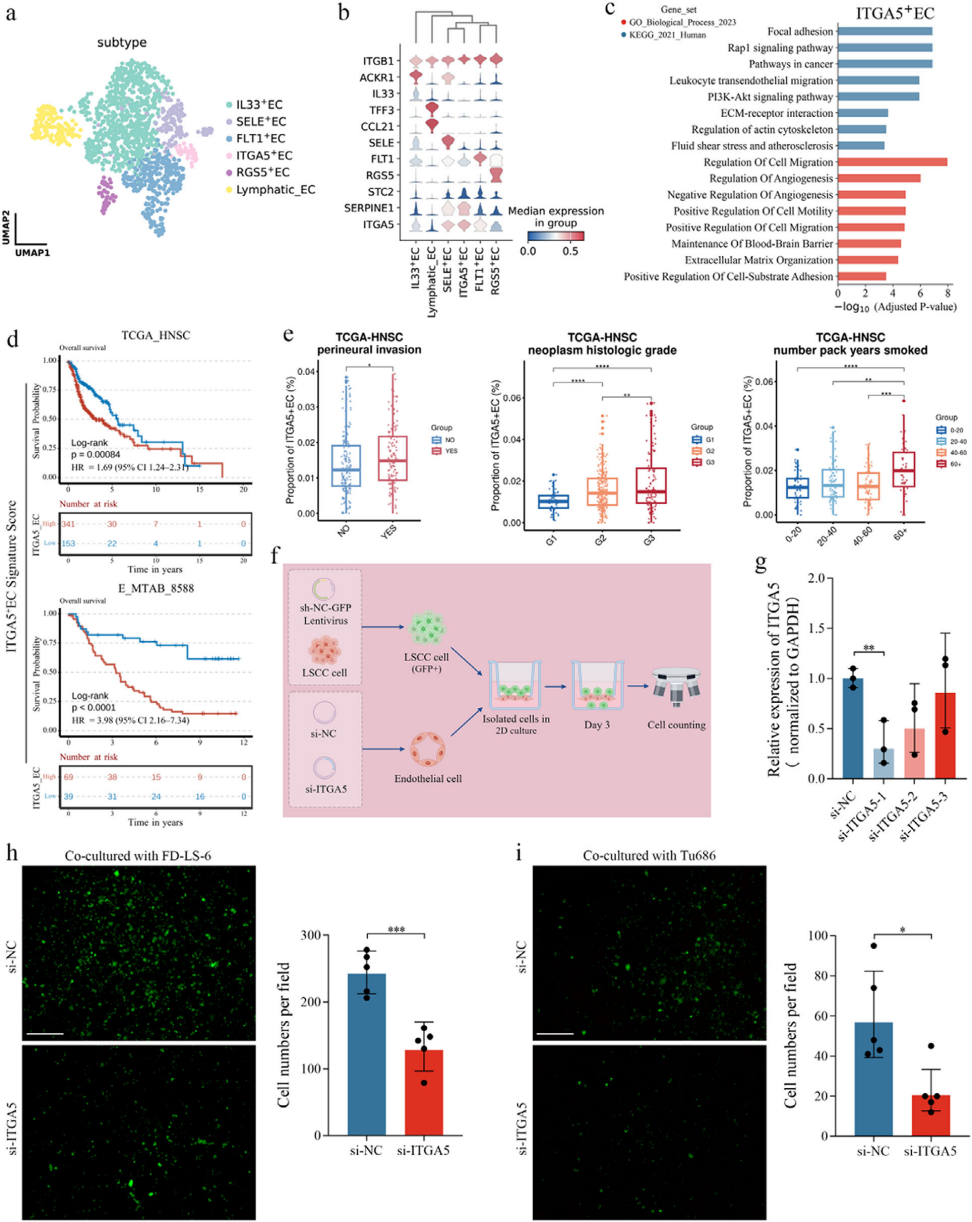
scRNA‐seq reveals ITGA5^+^ ECs and their biological function (a) Uniform manifold approximation and projection plot showing the classification of ECs, with distinct colors representing each subtype. b) Stacked violin plot depicting the expression levels of selected genes across the six EC subtypes. Color depth indicates the median expression in group. c) Representative KEGG and GO pathway enrichment analyses of marker genes expressed in ITGA5^+^ ECs (Hypergeometric test, BH‐adjusted *p* < 0.05). d) Kaplan–Meier curves for patients with HNSCC stratified by the signature scores of marker genes expressed in ITGA5^+^ ECs. Stratification was based on the optimal cutoff threshold, and *p*‐values were calculated using the Log‐rank test. e) Relative infiltration proportions of ITGA5^+^ ECs in tumor tissues from the TCGA‐HNSC cohort, stratified by perineural invasion, neoplasm histologic grade, and pack years smoked. Boxes represent the median ± interquartile range (IQR), with whiskers extending to the smallest or largest values within 1.5× IQR from the box boundaries. Significant pairwise comparisons, determined by Mann‐Whitney U tests, are indicated with asterisks: ^*^ *p* < 0.05, ^**^ *p* < 0.01, ^***^ *p* < 0.001, ^****^ *p* < 0.0001. f) Schematic diagram of the experimental workflow (by Figdraw). g) Confirmation of ITGA5 expression in ECs transfected with si‐NC, si‐ITGA5‐1, si‐ITGA5‐2, and si‐ITGA5‐3. GAPDH was used as a loading control. Data are presented as mean ± SD (n = 3). Statistical significance was determined using the unpaired *t*‐test, ^**^ *p* < 0.01. Number of FD‐LS‐6 h) and Tu686 i) cells transfected with control siRNA or ITGA5 siRNA that migrated across the endothelial monolayer. Data are presented as mean ± SD (n = 5). Statistical significance was determined using the unpaired *t*‐test, ^*^ *p* < 0.05, ^***^ *p* < 0.001.

The heterogeneity of ECs plays a critical role in tumor progression. After batch correction across patients, six distinct EC subpopulations were identified based on gene expression profiles: IL33^+^ ECs, SELE^+^ ECs, ITGA5^+^ ECs, RGS5^+^ ECs, FLT1^+^ ECs, and Lymphatic ECs (**Figure** **6**a; Figure S2a, Supporting Information). Among these, ITGA5^+^ ECs and FLT1^+^ ECs were more abundant in tumor tissues than in non‐tumor tissues, while Lymphatic ECs showed the opposite trend (Figure S2b–g, Supporting Information). In addition to differences in cell abundance, scRNA‐seq revealed distinct molecular features across EC subtypes. SELE^+^, ITGA5^+^, and FLT1^+^ ECs exhibited elevated expression levels of STC2, SERPINE1, and ITGA5 compared to other subtypes, with ITGA5^+^ ECs showing the highest average expression of all three genes (Figure 6b).

To explore the functional roles of EC subpopulations, pathway enrichment analysis was performed using GSEApy. Notably, ITGA5^+^ ECs were significantly enriched in tumor migration‐associated pathways (Figure 6c; Table S6, Supporting Information). Survival analysis of the top 15 markers of ITGA5^+^ ECs (Table S5, Supporting Information), ranked by log2FC, revealed their association with poor prognosis in the TCGA‐HNSC and E‐MTAB‐8588 cohorts (Figure 6d). Further analysis using CIBERSORTx deconvolution demonstrated that the abundance of ITGA5^+^ ECs correlated with perineural invasion, neoplasm histologic grade, and pack years smoked (Figure 6e), suggesting that ITGA5^+^ ECs contribute to malignant progression, particularly in the context of smoking exposure.

As presented in Figure 6c, ITGA5^+^ ECs were associated with transendothelial migration pathways. To further investigate the role of ITGA5 in ECs, in vitro experiments were conducted. ECs were transfected with either a non‐targeting control siRNA (si‐NC) or an ITGA5‐specific siRNA (si‐ITGA5) and co‐cultured with GFP‐labeled FD‐LS‐6 and Tu686 cells (Figure 6f). Efficient ITGA5 knockdown was confirmed by qRT‐PCR (Figure 6g), and ECs transfected with the si‐ITGA5‐1 construct were selected for subsequent assays. ITGA5 knockdown significantly reduced the number of FD‐LS‐6 (*p* = 0.0005, Figure 6h) and Tu686 (*p* = 0.0142, Figure 6i) cells that migrated through the endothelial monolayer from the upper to the lower chamber. These results indicate that silencing ITGA5 reduces EC permeability, thereby suppressing transendothelial migration and the hematogenous metastatic potential of LSCC cells.

To place these functional effects in a systems context, we first profiled cross‐lineage communication with CellChat,^[^ ^16^ ^]^ which identified epithelial tumor cells, myeloid cells, and fibroblasts as the dominant signaling sources to endothelial cells, with comparatively weaker contributions from T/NK and B cells (Figure S2h, Supporting Information). Then, we evaluated the putative crosstalk with the R package NicheNet^[^ ^17^ ^]^ based on the expression and downstream targets of ligand‐receptor pairs as an initial screen; we found that ITGA5^+^ endothelial cells and the STC2⁺ Tumor_C1 subpopulation emerged as reciprocal communication partners (Figure S2i–q, Supporting Information).

### 2.7 Spatial Transcriptomics Reveals Stc2+ Tumor_C1 Subpopulation Co‐Localized with Itga5+ Endothelial Cells in Smoking Lscc Samples

**Figure 7:**
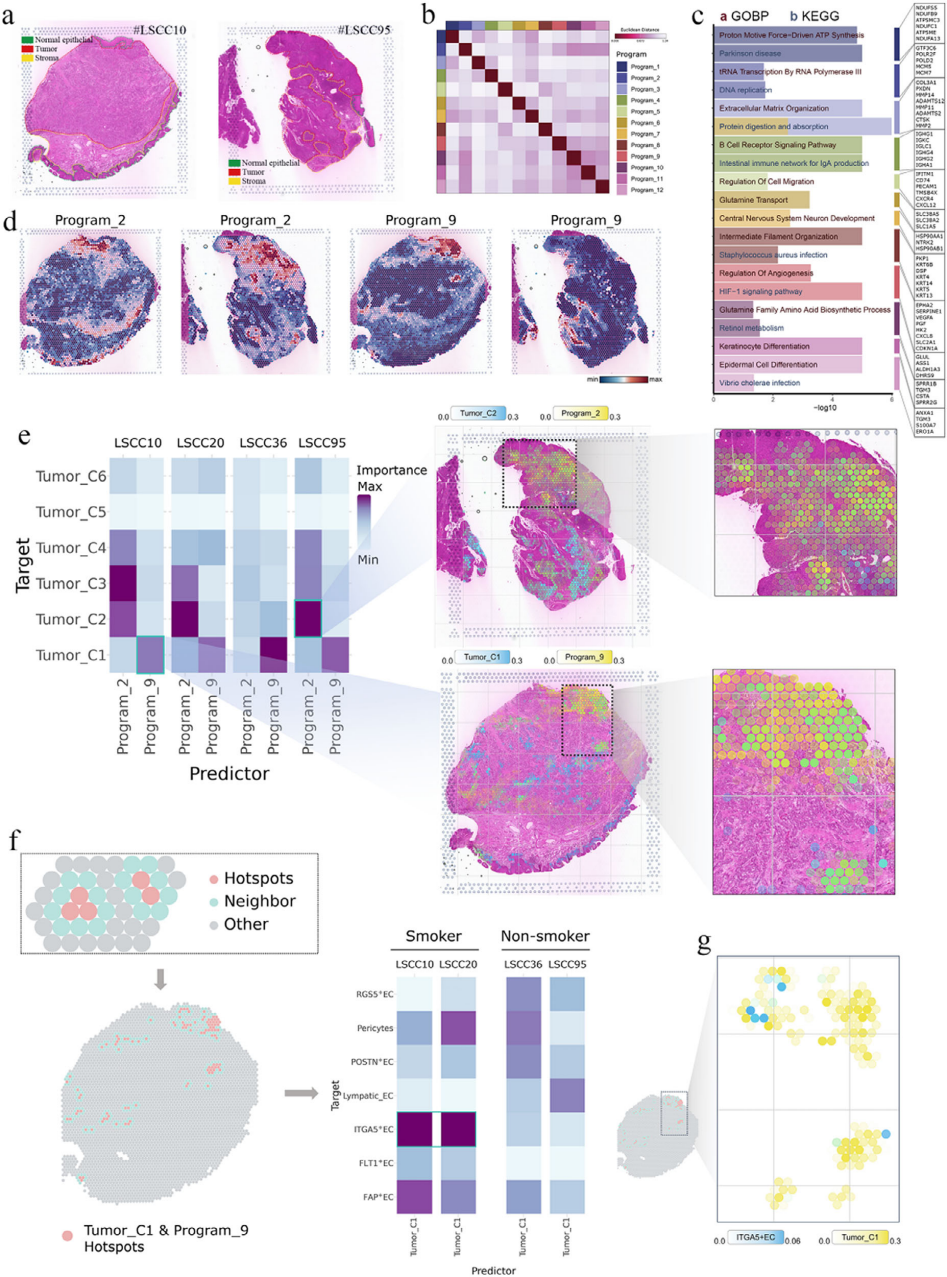
Spatial transcriptomic mapping reveals the interaction between STC2^+^ Tumor_C1 subpopulation and transcriptional Program 9 (a) H&E‐stained tissue sections from LSCC patients #10 (smoker) and #95 (non‐smoker) with overlaid Visium spatial transcriptomics spots. Tumor regions, stroma, and adjacent normal epithelium are outlined. b) Euclidean distance matrix showing transcriptional similarity among the 12 spatial gene programs identified by cNMF. c) GO and KEGG pathway enrichment analysis for the top 50 genes of each spatial program, highlighting significantly enriched terms (BH‐adjusted *p* < 0.05). d) Spatial feature plots illustrating Program 2 and Program 9 scores across the tumor sections. e) Heatmap displaying the predictive importance (MISTy weight) of each spatial program for epithelial subclusters. Corresponding tissue maps show co‐localization of Tumor_C2 with Program 2 and Tumor_C1 with Program 9 (color scales represent cell abundance or program score). f) Spatial map identifying “Tumor_C1 & Program_9 hotspots” (spots with top‐decile Tumor_C1 abundance and Program 9 score) and their neighboring locations. Heatmap showing the predictive importance (MISTy weight) of Tumor_C1 and Tumor_C2 for endothelial subclusters. g) Co‐distribution of Tumor_C1 cells and ITGA5^+^ ECs in the same tumor section, visualized by spatial feature mapping (color intensity indicates the relative abundance of Tumor_C1 cells or ITGA5^+^ ECs).

To further characterize the spatial architecture of tumor cells and ECs in LSCC, ST profiling was performed on resected LSCC tissues (**Figure** **7**a; Figure S3a, Supporting Information). In patient samples #10, #20, #36, and #95, ST identified 3700, 1732, 2180, and 2319 spots, respectively, with average sequencing depths ranging from 84 000 to 215 000 reads per spot and a median of 6900–7800 genes detected per spot. The cell2location algorithm was applied to deconvolve the cellular composition of each spot, mapping cell states defined by scRNA‐seq onto the spatial tissue context. Additionally, consensus non‐negative matrix factorization (cNMF) was used to identify 12 distinct gene expression “programs” from the ST data, each representing a set of co‐expressed genes (Figure 7b). The optimal number of components (k = 12) was determined through cNMF stability and error analysis (Figure S3b, Supporting Information). These spatial programs correspond to specific biological processes and cell‐type signatures (Figure 7c,d; Table S7, Supporting Information), including a DNA replication program (Program 2), an ECM remodeling program (Program 3), and an angiogenesis/HIF‐1 signaling program (Program 9).

MISTy^[^ ^18^ ^]^ was employed to explore the relationship between these gene programs and specific tumor subpopulations. This analysis revealed a strong association between the abundance of the Tumor_C1 cluster and angiogenic Program 9, suggesting a link between Tumor_C1 cells and pro‐angiogenic activity within the TME (Figure 7e). Spatially, both the Tumor_C1 cluster and Program 9 were predominantly localized at the tumor leading edge, a region associated with tumor invasion and metastasis. In contrast, the abundance of the Tumor_C2 cluster was most strongly correlated with Program 2, reflecting the highly proliferative nature of Tumor_C2 cells (Figure 7e). Further spatial mapping demonstrated that both the Tumor_C2 cluster and Program 2 were primarily confined to the central tumor regions, marked by elevated proliferative activity. Mechanistically, silencing STC2—a marker highly expressed by Tumor_C1—induced cytoskeletal remodeling via downregulation of small GTPases (RHOA, CDC42, RAC1) and their downstream effectors (PAK4, ROCK1), a pathway closely associated with angiogenesis.

**Figure 8:**
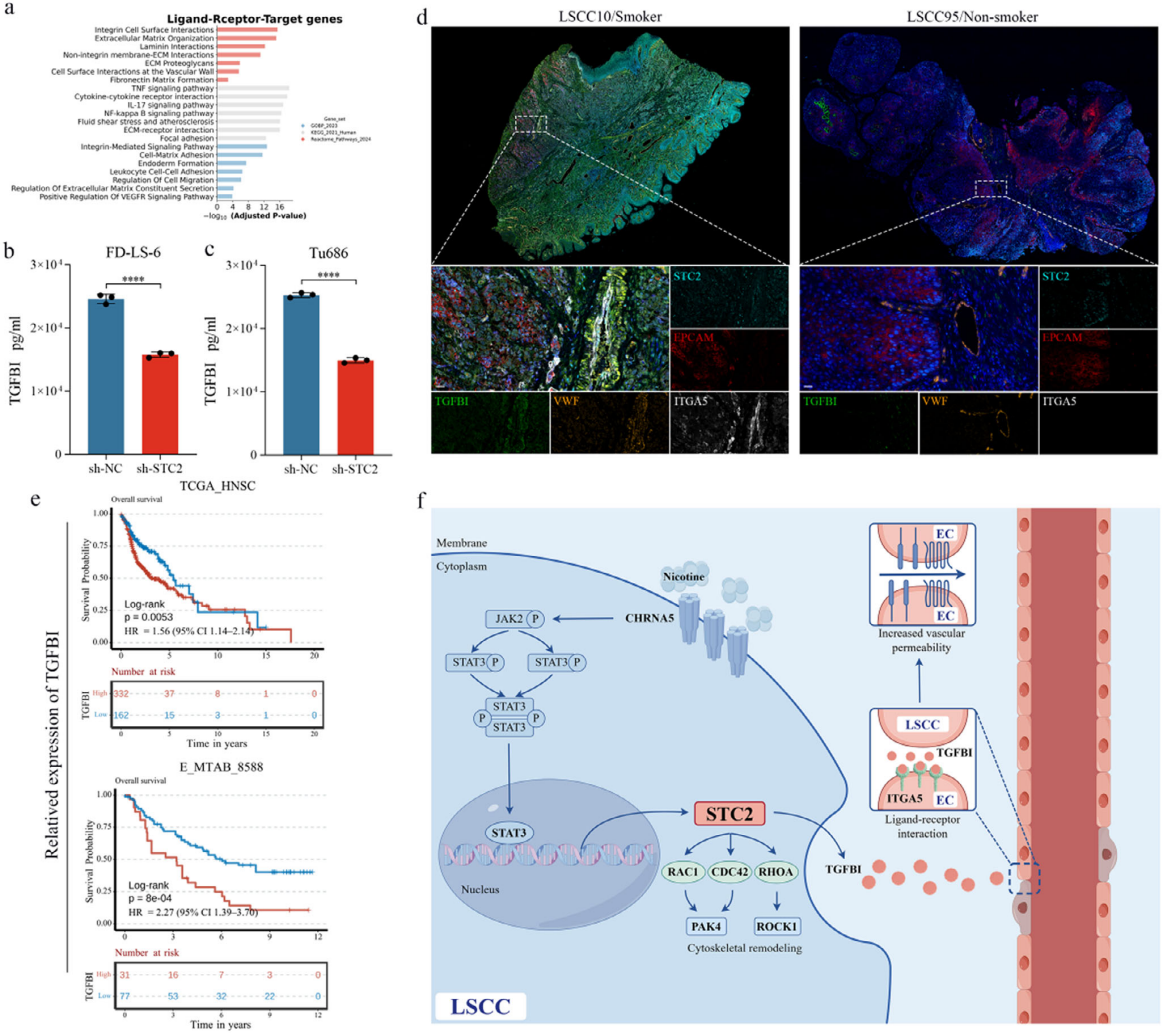
Ligand‐receptor crosstalk between STC2^+^ Tumor_C1 cells and ITGA5^+^ ECs (a) Representative GO, KEGG, and Reactome pathway enrichment of the top ligands expressed by Tumor_C1, receptors, and predicted target genes expressed by ITGA5^+^ ECs (Hypergeometric test, BH‐adjusted *p* < 0.05). Alteration of TGFBI protein levels in the supernatant of FD‐LS‐6 (b) and Tu686 (c) cells following STC2 knockdown. Data are presented as mean ± SD (n = 3). Statistical significance was determined using the unpaired t‐test, ^****^ *p* < 0.0001. d) Representative multiplex immunofluorescence staining results of smoking and non‐smoking LSCC tumor samples stained for STC2 (cyan), EPCAM (red), ITGA5 (white), VWF (orange), TGFBI (green), and DAPI (blue). Scale bar: 50 µm. e) Kaplan–Meier curves of patients with HNSCC stratified by TGFBI expression. The optimal cutoff threshold was used for stratification, with *p*‐values calculated using the Log‐rank test. f) Molecular mechanism underlying tumor‐vascular crosstalk reprogramming by the smoking‐induced STC2^+^ tumor cell subset (by Figdraw).

Building on these findings, this study examined tumor regions where Tumor_C1 cells, characterized by high STC2 expression, co‐localized with significant angiogenic activity. “Tumor_C1 & Program_9 hotspots” were defined as spatial spots in the top 90th percentile for both Tumor_C1 cell abundance and Program 9 score, with neighboring spots included for further analysis (Figure 7f). MISTy was employed to examine the spatial interdependence between Tumor_C1 hotspots and various EC subsets. In tumor sections from smoking patients, Tumor_C1 cells exhibited the strongest predictive association with ITGA5^+^ ECs (Figure 7f,g), indicating that these STC2‐high tumor clusters preferentially associate with and potentially interact with a specific angiogenic endothelial subpopulation. These results suggest that Tumor_C1 cells may actively promote angiogenesis by engaging ITGA5^+^ ECs, potentially influencing vascular permeability within the tumor. To further explore this interaction, ligand‐receptor mapping and pathway enrichment analyses were performed to identify the signaling axes through which Tumor_C1 might modulate ITGA5^+^ ECs (**Figure** **8**).

### 2.8 TGFBI–ITGA5 Ligand–Receptor Interaction Mediates STC2⁺ Tumor_C1–ITGA5⁺ Endothelial Cell Crosstalk in Smoking‐Associated LSCC

To identify key mediators of the interaction between ITGA5^+^ ECs and tumor cells in patients with LSCC, we provide a focused interpretation of the previous NicheNet results to delineate the specific ligand‐receptor pairs and predicted endothelial programs linking Tumor_C1 to ITGA5^+^ endothelial cells. Two approaches were compared: a sender‐agnostic approach, which does not predefine the sender cell type, and a sender‐focused approach, where all tumor cells were assigned as the ligand‐sending population. The results showed that most of the top‐ranked ligands identified by the sender‐agnostic method were also expressed in tumor cells, with only a few excluded in the sender‐focused approach due to a lack of expression (Figure S3c, Supporting Information). This suggests that tumor cells are likely the primary source of these highly active ligands. Notably, Tumor_C1 cells exhibited high expression of the majority of these ligands (Figure S2i,j, Supporting Information).

Tumor_C1 cells were found to directly interact with ITGA5^+^ ECs via adhesive ligand–receptor pairs, such as TGFBI–ITGA5, LAMA5/TGFBI–ITGA3, and ICAM1‐MSN/EZR (Figure S2k, Supporting Information). Furthermore, Tumor_C1 cells enhanced the pro‐inflammatory activity of ITGA5^+^ ECs by expressing TNF, TNFSF10, and inflammatory cytokines like IL1A and IL1B. These inflammation‐associated ligands induced the expression of CXCL8, VCAM1, SELE, TNFAIP3, and ICAM1 in ITGA5^+^ ECs (Figure S2l, Supporting Information). Many of these target genes are critical mediators of inflammatory endothelial activation and vascular remodeling. Among the 100 predicted targets, 42 encoded components involved in pro‐inflammatory signaling, leukocyte recruitment, and endothelial permeability regulation, including chemokines (e.g., CXCL2, CXCL3, CXCL8, CCL2, CCL3L1), adhesion molecules (e.g., ICAM1, VCAM1, SELE), and key NF‐κB pathway regulators (e.g., NFKB1, NFKB2, NFKBIA, NFKBIZ, REL, TNFAIP3, TNFAIP2, BCL3). Transcriptional regulators of endothelial stress responses were also enriched (e.g., FOS, JUN, JUNB, JUND, ATF3, EGR1, EGR3, KLF2, KLF4, KLF6), along with oxidative stress modulators (e.g., HMOX1, TXNIP, SOD2) and coagulation‐associated factors (e.g., F3, SERPINE1, CD55) (Figure S2l, Supporting Information).

A protein–protein interaction (PPI) network was constructed to illustrate the functional connectivity between the top ligands expressed by Tumor_C1 and receptor genes, along with target genes in ITGA5^+^ ECs (Figure S2m, Supporting Information). Additionally, enrichment analysis was conducted on the top ligands expressed by Tumor_C1, their corresponding receptors, and the predicted target genes expressed by ITGA5^+^ ECs, including GO, KEGG, and Reactome pathways (Figure 8a; Table S8, Supporting Information). The analysis revealed that the enriched pathways were strongly associated with inflammatory signaling and ECM remodeling. Specifically, the target genes were enriched in pathways such as cytokine–cytokine receptor interaction, TNF signaling, NF‐kappa B signaling, ECM–receptor interaction, and integrin‐mediated signaling. Furthermore, pathways related to vascular regulation and remodeling, including fibronectin matrix formation, laminin interactions, and cell surface interactions at the vascular wall, were prominently represented. These findings suggest that the interaction between Tumor_C1 and ITGA5^+^ ECs may enhance vascular permeability. ELISA results showed that STC2 knockdown significantly reduced TGFBI secretion by LSCC cells (*p* < 0.0001, Figure 8b,c). Multiplex immunofluorescence staining revealed that STC2 was expressed in EPCAM^+^ LSCC tumor cells, while ITGA5 was specifically localized to VWF^+^ vascular ECs and co‐localized with TGFBI (Figure 8d). Kaplan–Meier survival analysis indicated that high TGFBI expression was associated with poorer overall survival (OS) (Figure 8e). Notably, this cell‐type‐specific expression pattern was observed exclusively in smoking patients with LSCC, suggesting that tobacco exposure may promote tumor‐endothelial crosstalk in LSCC via the STC2–TGFBI–ITGA5 signaling axis (Figure 8f).

## 3 Discussion

Nicotine‐nAChR‐STAT3 signaling^[^ ^19^, ^20^, ^21^, ^22^, ^23^, ^24^, ^25^ ^]^ and TGFBI–integrin biology have been explored in various cancers,^[^ ^26^, ^27^, ^28^, ^29^, ^30^, ^31^ ^]^ but their convergence and spatial organization in smoking‐associated LSCC remained unclear. In previous research, nicotine‐induced activation of CHRNA5 triggered the JAK2/STAT3 signaling cascade, enhancing the metastatic potential of LSCC.^[^ ^13^ ^]^ However, these studies did not define a transcriptionally unified, smoking‐enriched tumor subset or establish a link between STAT3 and transcriptional regulation of STC2 in LSCC cells.

This study presents, for the first time, a comprehensive spatial and single‐cell transcriptomic analysis of 13 distinct cellular subpopulations in smoking‐associated LSCC samples. Notably, smoking‐associated prognostic gene signatures, including STC2 and ITGA5, were predominantly enriched in epithelial cells and ECs, suggesting that tobacco exposure may promote LSCC progression by epigenetically reprogramming specific stromal cell phenotypes.

Through integrative analysis of single‐cell and ST data, a previously unrecognized bifurcation of STC2‐expressing tumor cells into two distinct subpopulations was identified: a quiescent Tumor_C1 phenotype and a proliferative Tumor_C2 phenotype, highlighting the functional plasticity of STC2 within the TME. Spatial mapping revealed a correlation between the Tumor_C1 subpopulation and an angiogenesis‐associated transcriptional program (Program_9). Core driver genes of Program_9, including EPHA2, SERPINE1, VEGFA, PGF, HK2, CXCL8, SLC2A1, and CDKN1A, suggest that these regulators—particularly VEGFA^[^ ^32^ ^]^—which promote EC proliferation and migration, contribute to the STC2–TGFBI–ITGA5 signaling axis, potentially enhancing vascular permeability and facilitating hematogenous metastasis. In contrast, the Tumor_C2 subpopulation was linked to a DNA replication program (Program_2), suggesting a role in tumor stemness and proliferative capacity.

Clinically, elevated STC2 expression strongly correlated with tumor grade, TNM stage, degree of differentiation, and OS in patients with HNSCC, supporting its potential as a novel smoking‐associated prognostic biomarker. These findings reinforce previous reports of STC2's pro‐metastatic roles in other malignancies,^[^ ^33^, ^34^, ^35^ ^]^ despite observed tissue‐specific differences in expression and function. To further investigate STC2's biological role, stable overexpression cell lines were generated via lentiviral transduction. Both in vitro and in vivo assays demonstrated that STC2 enhanced LSCC cell proliferation, migration, and invasion.

Mechanistically, this study is the first to identify STAT3 as a transcriptional regulator of the STC2 promoter and to describe a nicotine–CHRNA5–JAK2/STAT3–STC2 signaling cascade in LSCC. The spatial expression patterns of the CHRNA5–JAK2/STAT3–STC2 axis indicate its predominant activation in smoking‐associated LSCC (Figure S4, Supporting Information). Additionally, STC2 knockdown was found to modulate F‐actin cytoskeletal reorganization through small GTPase signaling in smoking‐associated LSCC (Figure S5, Supporting Information), impairing pseudopodia formation and restoring epithelial polarity, thus enhancing our understanding of how STC2 drives tumor cell motility and migration at the molecular level.

ITGA5, a critical mediator of cell‐surface adhesion and intracellular signaling, plays pivotal roles in malignancies.^[^ ^36^, ^37^, ^38^, ^39^, ^40^, ^41^, ^42^ ^]^ In this study, the abundance of ITGA5^+^ ECs was positively correlated with perineural invasion, higher histological grade, increased smoking pack‐years, and poor prognosis in patients with HNSCC, suggesting that these cells are key components of a pro‐metastatic niche.

Functionally, silencing ITGA5 in vitro reduced endothelial permeability and impaired the trans‐endothelial migration of LSCC tumor cells, a phenotype consistent with previous studies on ITGA5‐mediated vascular mimicry.^[^ ^43^ ^]^ Notably, this study is the first to reveal, through integrated single‐cell and ST analyses, potential cellular crosstalk between ITGA5^+^ ECs and STC2^+^ Tumor_C1 subpopulations, suggesting a coordinated role in driving tumor progression and metastasis.

TGFBI is an ECM protein regulated by the TGF‐β signaling pathway,^[^ ^14^ ^]^ known for its complex roles in tumor proliferation, angiogenesis,^[^ ^44^ ^]^ and metastasis.^[^ ^45^, ^46^ ^]^ The impact of TGFBI on tumor angiogenesis exhibits dichotomous effects across various tumor types. On one hand, the FAS1 structural domain of TGFBI has been shown to exert anti‐angiogenic effects both in vitro and in vivo by directly interacting with αvβ3 integrins,^[^ ^47^, ^48^ ^]^ thereby contributing to tumor suppression, particularly in melanoma. On the other hand, TGFBI has been reported to promote angiogenesis and metastasis in certain tumors, such as colorectal cancer,^[^ ^49^ ^]^ suggesting a pro‐tumorigenic function. Several recent reviews and mechanistic studies emphasize that these apparently contradictory roles are determined by the cellular source of TGFBI (tumor cell vs stromal/TAMs), the repertoire of integrins expressed by neighbouring endothelial or stromal cells (e.g., αvβ3/αvβ5 versus α5β1), post‐translational processing of TGFBI, and local microenvironmental cues such as hypoxia and TGF‐β activity.^[^ ^14^, ^27^ ^]^ In this study, TGFBI interacts with ITGA5 on endothelial cells and was associated with increased vascular permeability and dissemination, a result that is mechanistically concordant with pro‐angiogenic/pro‐permeability signalling mediated by α5β1 integrins in other systems.^[^ ^50^ ^]^ Thus, rather than contradicting prior reports, our data suggest that STC2/TGFBI signaling promotes a pro‐permeability/pro‐dissemination program in the specific cellular and integrin context of smoking‐associated LSCC.

Nicotine exposure, induced by smoking, facilitates the transendothelial migration of LSCC cells through the activation of the STC2–TGFBI–ITGA5 signaling axis in our study. Western blotting demonstrated an increase in STC2 expression following nicotine exposure (Figure 3e), accompanied by a significant elevation of TGFBI levels in the culture supernatant (Figure S3h,i, Supporting Information) and ITGA5 in ECs (Figure S3d, Supporting Information), suggesting that nicotine induces STC2 upregulation in parallel with enhanced TGFBI secretion. STC2 silencing markedly decreased TGFBI secretion (Figure 8b,c). In functional assays, HUVEC monolayers (80% confluence) were cultured with tumor cell supernatants (sh‐NC or sh‐STC2; 1:1 supernatant:ECM) for 72 h, followed by FITC‐dextran treatment (250 µg mL^−1^). Compared with sh‐NC, the sh‐STC2 group showed markedly reduced FITC‐dextran fluorescence intensity (Figure S3e,f, Supporting Information), indicating suppressed endothelial permeability. ST and multiplex immunofluorescence further confirmed the interaction between TGFBI and ITGA5, suggesting that tumor‐derived TGFBI may enhance endothelial permeability by activating ITGA5 expression in ECs. This tumor‐endothelial crosstalk appears to be particularly prominent in smoking‐associated LSCC (Figure 8d). FITC‐dextran assays demonstrated higher fluorescence intensity in ECs exposed to nicotine‐conditioned medium compared with controls (Figure S3j,k, Supporting Information). Importantly, this effect was abolished by TGFBI knockdown (Figure S3g, Supporting Information) and restored upon nicotine stimulation (Figure S3j,k, Supporting Information), confirming that TGFBI mediates nicotine‐enhanced endothelial permeability. Knockdown of ITGA5 in ECs reduced the number of FD‐LS‐6 (Figure 6 h) and Tu686 (Figure 6i) cells that migrated through the endothelial monolayer from the upper to the lower chamber. To address potential concerns regarding sample size and patient heterogeneity, multiplex immunofluorescence staining was performed on a tissue microarray comprising 61 pairs of LSCC tumors and matched adjacent normal tissues (Figure S6a, Supporting Information; detailed clinical information provided in Table S9, Supporting Information), which were obtained from the Eye and ENT Hospital, Fudan University. Consistent with the findings in Figure 8d, smoking‐associated LSCC specimens (Figure S6b, Supporting Information) exhibited elevated expression of STC2, TGFBI, and ITGA5 in tumor tissues compared with their paired adjacent normal epithelia. Notably, ITGA5 was specifically localized to VWF⁺ vascular endothelial cells and colocalized with TGFBI. In contrast, these patterns were absent in LSCC specimens from non‐smoking patients (Figure S6c, Supporting Information). Additionally, independent validation using the TCGA‐LSCC cohort (n = 111) confirmed consistent overexpression of STC2, TGFBI, and ITGA5 in LSCC tumor tissues and their strong association with adverse patient outcomes (Figure S7, Supporting Information). Notably, stratification by smoking status further demonstrated that these genes were specifically enriched and prognostically unfavorable in the smoking‐associated LSCC cohort (n = 102, Figure S8, Supporting Information).

This study has several limitations. First, although the patients shared highly similar clinical characteristics (male current smokers, aged 67–72 years, with LSCC, staged T3–T4a, N0, M0, Table S1, Supporting Information) and per‐patient UMAP analyses confirmed consistent clustering patterns across individuals (Figure S1c, Supporting Information), the sample sizes of both the scRNA‐seq and ST datasets were modest. Expanding the number of profiled patients and spatial sections would better capture the heterogeneity of smoking‐associated subpopulations and increase power to resolve cell‐cell spatial associations. Second, models of pulmonary colonization are necessary to evaluate the effect of STC2 knockout on the hematogenous metastatic potential of LSCC cells. Third, although our findings provide evidence that STC2 promotes TGFBI secretion and subsequently enhances ITGA5‐dependent endothelial permeability, we acknowledge that this mechanism may not be exclusive. Other downstream effectors of STC2, including cytoskeletal regulators and extracellular matrix proteins, may also contribute to the observed effects on endothelial dynamics and tumor dissemination. Finally, the potential of targeted therapies—such as TGFBI or ITGA5 inhibitors—to disrupt LSCC metastasis and trans‐endothelial migration warrants further investigation.

Through a multi‐omics integrative approach, this study systematically outlined the molecular framework by which smoking reshapes the TME in LSCC. For the first time, a dual‐subtype STC2^+^ tumor cell population was identified as a central mediator of both tumor cell motility and tumor–EC crosstalk. These findings provide novel mechanistic insights and highlight potential therapeutic targets for prognostic stratification and precision treatment of smoking‐associated LSCC.

## 4 Experimental Section

### Patient Specimens and Ethics Statement

A total of nine LSCC specimens, including three from proximal cancerous tissue, three from the junction area, and three from lateral cancer‐adjacent tissue, were obtained from LSCC patients for scRNA‐seq. In addition, four LSCC specimens (two from smoking and two from no‐smoking LSCC patients) were collected for ST and multiplex immunofluorescence staining. All tissue samples were obtained from patients undergoing surgical treatment at the Department of Otorhinolaryngology, Eye and ENT Hospital of Fudan University (Table S1, Supporting Information). The ethics committee of the Eye and ENT Hospital of Fudan University approved the study protocols (approval number: 2021039). Written informed consent was obtained from all patients prior to enrollment.

### Preparation of Single‐Cell Suspension

Fresh tumor tissues were obtained intraoperatively from LSCC patients. Under sterile conditions, the tissues were washed twice with pre‐chilled RPMI 1640 medium supplemented with 0.04% BSA. The samples were then finely minced into ∼0.5 mm^3^ pieces using sterile surgical scissors and transferred into freshly prepared digestion buffer containing 0.2% collagenase type II (17101015, Gibco). Tissue digestion was performed at 37 °C in a humidified incubator for 30 min, with gentle agitation every 5–10 min to ensure adequate dissociation.

The resulting cell suspension was filtered through a 40 µm cell strainer (352340, Falcon) one to two times to remove undigested fragments, followed by centrifugation at 300×g for 5 min at 4 °C. The cell pellet was resuspended in an appropriate volume of culture medium and mixed with an equal volume of red blood cell lysis buffer (130‐094‐183, Miltenyi Biotec), then incubated at 4 °C for 10 min. The suspension was centrifuged again at 300×g for 5 min, and the supernatant was discarded. The pellet was washed once with culture medium, centrifuged again under the same conditions, and finally resuspended in 100 µl of medium. Cell concentration and viability were assessed using an automated cell counter (Luna‐FL, Logos Biosystems).

### Library Preparation, Sequencing, and Data Acquisition

Freshly prepared single‐cell suspensions were adjusted to a final concentration of 700–1200 cells µL^−1^. Library preparation was performed using the 10×Genomics Chromium Next GEM Single Cell 3ʹ Reagent Kits v3.1 (Cat. No. 1000268, 10× Genomics), following the manufacturer's protocol. The resulting libraries were subjected to high‐throughput sequencing on the Illumina NovaSeq 6000 platform with 150 bp paired‐end reads (PE150).

### Visium CytAssist Spatial Transcriptome Sequencing

A total of four tissue sections derived from four independent LSCC patients undergoing surgical treatment at the Department of Otorhinolaryngology, Eye and ENT Hospital of Fudan University were analyzed. Detailed clinicopathological information for each sample was comprehensively summarized in Table S1 (Supporting Information). Freshly excised LSCC tissues were sectioned into appropriately sized blocks, immediately fixed in 4% paraformaldehyde (PFA), and embedded in paraffin. Tissue sections were mounted onto Visium CytAssist‐compatible slides in accordance with the 10x Genomics protocol. Deparaffinization, hematoxylin and eosin (H&E) staining, imaging, and decrosslinking were performed following the manufacturer's instructions (CG000520). Subsequent probe hybridization, probe release, and library preparation were conducted using the Visium CytAssist Spatial Gene Expression for FFPE kit (PN‐1000520 for Human, 6.5 mm) according to the protocol CG000495. Final libraries were subjected to high‐throughput paired‐end 150 bp (PE150) sequencing.

### ScRNA‐Seq and Differential Expression Analysis

Sequencing reads were aligned and gene expression quantified using Cell Ranger (v5.0.0, 10x Genomics). Subsequent analyses were conducted with Scanpy (v1.11.1) in Python. Quality control proceeded as follows. At the gene level, genes detected in at least 3 cells were retained. At the cell level, n_genes_by_counts between 500 and 6000, total UMI counts ≥ 1000, and mitochondrial gene fraction ≤ 15% (mitochondrial genes identified by the “MT‐” prefix) were required. Putative doublets were identified in DoubletFinder (v2.0.3, R) on raw counts and the doublet labels were mapped back to AnnData; flagged cells were removed.

To mitigate batch effects and minimize inter‐individual variability, data integration across samples was performed using BBKNN. Highly variable genes were identified using scanpy.pp.highly_variable_genes, selecting the top 3106 genes based on expression variability. The data were then normalized and scaled accordingly. Principal component analysis (PCA) was conducted on the selected highly variable genes. A neighborhood graph was constructed using the top 30 principal components with scanpy.pp.neighbors. Subsequent clustering was performed using the Leiden algorithm (scanpy.tl.leiden), and the results were visualized using Uniform Manifold Approximation and Projection (UMAP).

Differential gene expression analysis between clusters was carried out using the Wilcoxon rank‐sum test via scanpy.tl.rank_genes_groups. Multiple testing correction was applied using the Benjamini‐Hochberg procedure to control the false discovery rate.

### Functional Enrichment and Gene Set Enrichment Analysis

Functional enrichment analyses, including Kyoto Encyclopedia of Genes and Genomes (KEGG), Gene Ontology (GO), and Reactome pathways, as well as gene set enrichment analysis (GSEA), were performed using the GSEApy Python package (v1.13.1). Pathways with adjusted *p* < 0.05 were considered significantly enriched.

### Quantification of Cell–Cell Communication

Cell–cell communication and interaction weights between different major cell types were quantified using CellChat. Cell‐cell communication between ITGA5⁺ ECs and tumor cells was inferred using NicheNet. Only ligands and receptors expressed in more than 5% of cells within each cluster were considered. The top 30 differentially expressed ligands from sender cells and the top 100 target genes from receiver cells were selected for ligand‐receptor activity analysis.

### Cell Type Deconvolution of Bulk RNA‐Seq Data

To assess the functional contributions of individual cell types across large‐scale samples, CIBERSORTx was employed, utilizing scRNA‐seq data to construct a reference signature matrix (S‐mode, quantile normalization disabled). This matrix was then applied to deconvolute bulk RNA‐seq datasets, estimating the relative proportions of constituent cell types. During the construction of the signature matrix, quantile normalization was disabled to accommodate the characteristics of RNA‐seq data, while other parameters were maintained at their default settings. During the deconvolution process, quantile normalization was disabled, and the number of permutations was set to 100 to ensure robust estimation.

### Inference of Epithelial Cell States Using inferCNVpy

To investigate copy number variations (CNVs) within epithelial cells, the inferCNVpy tool (v0.6.0) was applied to scRNA‐seq data. Epithelial cells derived from normal samples served as external references to facilitate the identification of large‐scale chromosomal alterations in tumor epithelial cells.

### Spatial Transcriptomic Data Analysis

Spatial transcriptomic data were processed using the Scanpy package (v1.11.1) in Python (v3.11). Initial quality control steps included retaining genes expressed in at least three cells and filtering out spatial spots with fewer than 300 detected genes or fewer than 600 UMIs. Cell‐type deconvolution was performed using Cell2location (v0.1.4), which estimated the abundance of each cell type across spatial spots based on reference single‐cell RNA‐seq data.

To identify spatially variable gene expression programs, consensus non‐negative matrix factorization (cNMF) was applied across all ST samples. The top 1500 spatially variable genes were selected based on Moran's I statistic. cNMF was executed with the number of factors (k) ranging from 5 to 15, and the optimal k (k = 12) was determined. Each program was annotated by examining the top 50 genes with the highest weights and performing gene set enrichment analysis using GSEApy.

### Modeling Spatial Dependencies with Multiview Intercellular SpaTial Modeling Framework (MISTy)

To assess the influence of malignant epithelial cells on the abundance of fibroblast subpopulations, the mistyR package (v1.10.0) was utilized. This multiview framework models spatial dependencies by constructing three distinct views: 1) the intrinsic view, capturing associations within individual spots; 2) the juxta view, summarizing information from neighboring spots within a radius of five spots; and 3) the para view, incorporating information from more distant spots within a radius of fifteen spots. By analyzing these views, the spatial relationships between malignant epithelial cells and fibroblast subtypes were evaluated, identifying patterns of co‐localization or mutual exclusivity.

### Cell Culture and Lentiviral Transduction

Three LSCC cell lines and one normal laryngeal epithelial cell line (HuLa‐PC) were utilised as previously described.^[^ ^13^ ^]^ FD‐LSC‐1, FD‐LS‐6, and HuLa‐PC were cultured in BEGM (CC‐3170, Lonza), while Tu686 was cultured in RPMI‐1640 (Gibco). For stable cell transfection, sh‐NC and sh‐STC2 (PGMLV‐hU6‐MCS‐CMV‐ZsGreen1‐PGK‐Puro‐WPRE) lentivirus was purchased from Genomeditech (Shanghai, China), and the lentiviral vectors were transfected into LSCC cells according to the manufacturer's instructions. The target sequences of sh‐NC, sh‐STC2, and si‐TGFBI are listed in Table S2 (Supporting Information).

### RNA Isolation and Quantitative Reverse Transcriptase PCR

RNA isolation and quantitative reverse transcriptase PCR were utilized as previously described.^[^ ^13^ ^]^ The TRIzol method was used for RNA extraction from fresh samples and LSCC cells. Evo M‐MLV RT Kit with gDNA Clean for qPCR (AG11711, Accurate Biotechnology (Hunan) Co., Ltd.) was applied for reverse transcription. qRT‐PCR was conducted using SYBR Green Premix Pro Taq HS qPCR Kit (AG11701, Accurate Biotechnology (Hunan) Co., Ltd.) for mRNA with ABI 7500 Real‐Time PCR System (Life Technologies, Shanghai, China), and the relative expression level was calculated with the 2^−ΔΔCt^ method. The sequences of all primers are listed in Table S3 (Supporting Information).

### Western Blotting

Western blotting was utilized as previously described.^[^ ^13^ ^]^ Proteins were extracted with 1× cell lysis buffer. The SDS‐PAGE electrophoresis and immunoblotting were performed as standard procedures with antibodies specific for STC2 (60063‐1‐lg, Proteintech), ITGA5 (ab150361, Abcam), RHOA (10749‐1‐AP), ROCK1 (21850‐1‐AP), CDC42 (10155‐1‐AP), RAC1 (66122‐1‐lg), and PAK4 (14685‐1‐AP). GAPDH was used as a control. Antibody details are in Table S4 (Supporting Information).

### Immunofluorescence Staining

Immunofluorescence staining was performed as previously described.^[^ ^13^ ^]^ Samples were fixed, permeabilized, and blocked before incubation with primary antibodies at 4 °C overnight. After incubation with fluorescent secondary antibodies, nuclei were stained with DAPI. Images were captured by fluorescence microscopy, and signal intensity was quantified using ImageJ.

### Immunohistochemistry

Immunohistochemistry was performed as previously described.^[^ ^13^ ^]^ Sections were processed for antigen retrieval, blocked, and incubated with primary antibodies at 4 °C overnight. After secondary antibody incubation, staining was developed with DAB.

### Chromatin Immunoprecipitation (ChIP)

ChIP assays were performed to assess the binding of STAT3 to the STC2 promoter. Briefly, HEK293T cells were crosslinked with 1% formaldehyde to covalently stabilize protein–DNA interactions, followed by cell lysis. Chromatin was enzymatically digested and further sheared by sonication to obtain DNA fragments of appropriate size. The chromatin was then immunoprecipitated with an anti‐STAT3 antibody or rabbit IgG as a negative control. Protein–DNA complexes were subsequently purified, and the associated DNA fragments were analyzed by quantitative PCR (qPCR) using specific primers targeting the STC2 promoter region (forward: 5′‐ACTGGGAAAGCATTTGGGGAGC‐3′; reverse: 5′‐GGGTGATGATGTTGGTGAAGGGT‐3′).

### Dual‐Luciferase Reporter Assay

Dual‐luciferase reporter assay was performed as previously described.^[^ ^15^ ^]^ Luciferase reporter plasmids incorporating mutant or wild‐type STAT3 binding sites within the STC2 promoter were constructed. HEK293T cells were transfected with the STAT3‐pcDNA3.1 plasmid and co‐transfected with wild‐type or mutant STC2 pGL3 plasmid using Lipofectamine 2000 reagent (Thermo Fisher). Luciferase activity was measured 48 h after transfection.

### Cell Viability Assay

Cell Counting Kit‐8 (CCK8) assay was applied to assess cell viability of LSCC cell lines as previously described.^[^ ^13^ ^]^ LSCC cells were spread in 96‐well plates (2 × 10^3^ cells per well). After cell attachment, 10 µL CCK8 and 90 µL basal medium were added to each well for 1 h. The absorbance at 450 nm was measured for five consecutive days using the Model 680 Microplate Reader (Bio‐Rad).

### Wound Healing Assay

Wound healing assay was performed as previously described.^[^ ^13^ ^]^ The LSCC cells were seeded into 6‐well plates and allowed to proliferate to 100% confluency. Artificial gaps were generated by scratching with a sterile 200 µL pipette tip. Then, the complete culture medium was replaced with serum‐free culture medium. Areas of the wound were marked and photographed at 0 and 48 h with an Olympus microscope (Tokyo, Japan), and measured by the “MRI Wound Healing Tool” plug‐in unit of Image J.

### Transwell Assay

The Transwell assay was performed as previously described.^[^ ^13^ ^]^ For migration assays, 600 µL complete culture medium was added to the lower chamber, and 1 × 10^5^ LSCC cells were added to 100 µL serum‐free culture medium in the upper chamber with an 8 µm pore size (Corning, New York, USA). After 72 h of incubation, the inserts were fixed with 4% methanol and then stained with 1% crystal violet. The stained cells were photographed with an Olympus microscope (Tokyo, Japan) and measured by Image J. For invasion assays, the inserts of the chambers in which the cells were seeded were coated with Matrigel (BD Biosciences).

### Xenograft Studies

To evaluate cell proliferation in vivo, six‐week‐old male nude mice (Chengqin Bio‐Technology (Shanghai) Co., Ltd.) were subcutaneously injected with 1 × 10^6^ sh‐NC or sh‐STC2 cells, respectively. The xenograft tumors were resected and weighed after the nude mice were sacrificed. The xenograft tumors were then fixed with 4% PFA for further immunohistochemical staining. All animal experiments were performed in compliance with the guidelines and authorization of the animal center at the Eye & ENT Hospital of Fudan University.

### Statistical Analyses

The detailed statistical test methods, sample sizes, and *p*‐values used in this study were indicated in the corresponding legends and captions. Statistical analyses were conducted using GraphPad Prism 7 (GraphPad Software, Inc., USA), R (v4.3.1), and Scanpy (v1.11.1) in Python 3.11. All results were presented as the mean ± standard deviation (SD). Student's t‐test was used to compare two groups when the data followed a normal distribution; otherwise, the nonparametric Mann‐Whitney U test was used. Kaplan‐Meier survival curves were generated and compared using the Log‐rank test. Univariate and multivariate Cox regression analyses were performed to identify independent risk factors. Variables with *p* < 0.05 in univariate analyses were entered into the multivariate model. A *p*‐value < 0.05 was regarded as statistically significant.

## Conflict of Interest

The authors declare no conflict of interest.

## Author Contributions

Y.J.S., L.C.G., and Q.H. contributed equally to this work. Y.J.S., L.C.G., and Q.H. designed the experiments. L.Z. contributed to executing the described experiments. Y.J.S. and L.C.G. were responsible for the bioinformatic analysis of the data. C.Z.X. analyzed the data. C.P.W contributed to the preparation of this manuscript, and all authors aided in editing the final manuscript.

## Supporting information



Supporting Information

## Data Availability

The Visium and scRNA‐seq data generated in this study have been deposited in the Genome Sequence Archive (accessions HRA011855 and HRA011830) and are publicly accessible at https://ngdc.cncb.ac.cn/gsa. The RNA‐seq TCGA‐HNSC and TCGA‐LSCC data used in this study are available in the TCGA data portal (https://portal.gdc.cancer.gov/). Access for non‐profit research will be approved upon application, and all other raw data are available from the corresponding author upon reasonable request. In addition, scripts used to generate the figures and the annotated scRNA‐seq datasets used in this study are available at https://github.com/LCGaoZzz/HNSCC_code.
