# Supplementary material for: Smoking‐Induced STC2+ Tumor Cells Drive Tumor‐Vascular Crosstalk in Laryngeal Squamous Cell Carcinoma via Spatial and Single‐Cell Transcriptomics
Source: Adv Sci (Weinh). 2025 Nov 7;13(5):e11932. doi: 10.1002/advs.202511932 (PMC12849985; doi:10.1002/advs.202511932)
Supplement: Supplementary file 1 — Supporting Information [file ADVS-13-e11932-s001.docx]

**
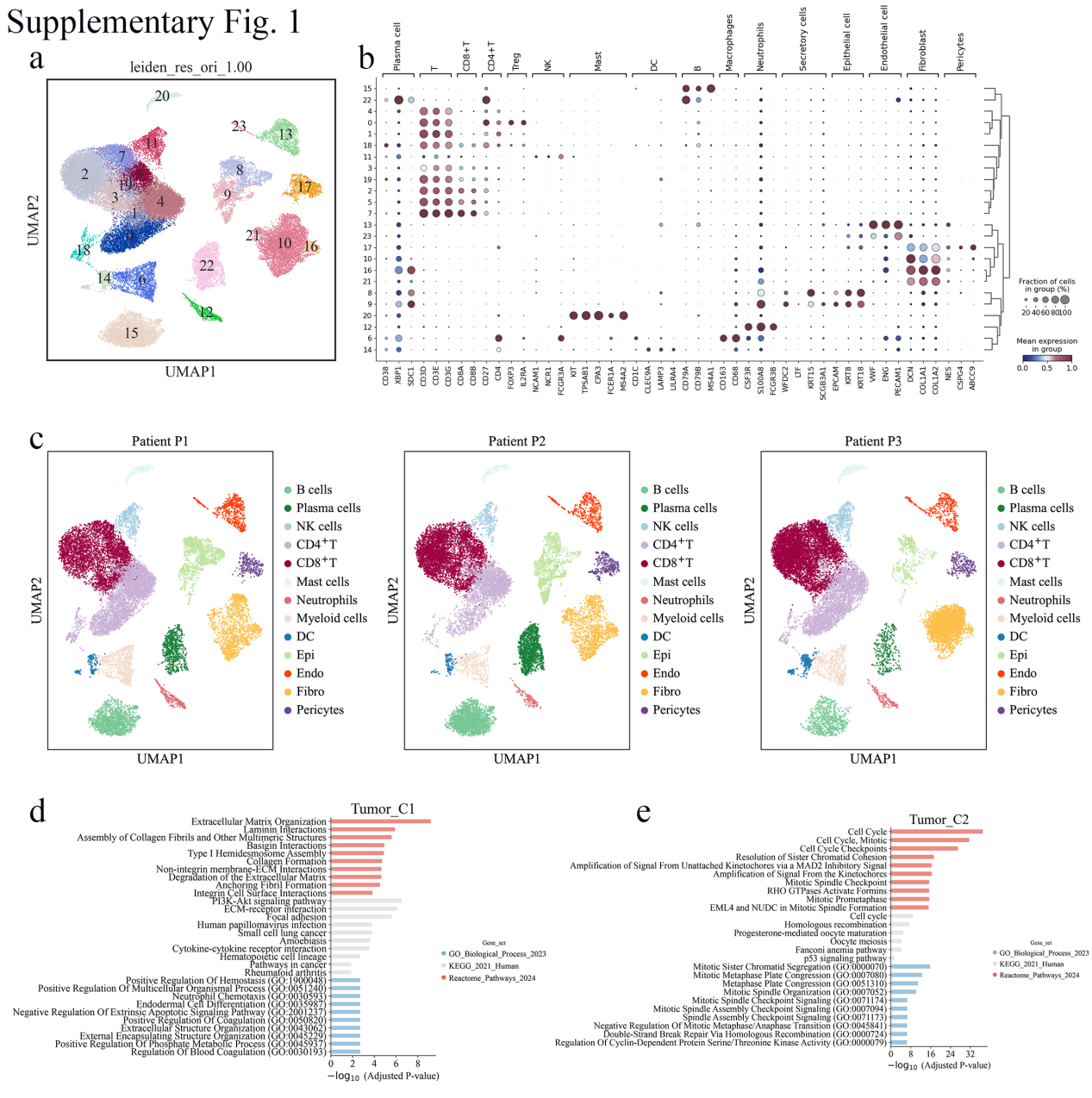
Supplementary Fig. 1 Leiden analysis reveals the main clusters of LSCC based on gene expression**(a) Leiden clustering depiction based on neighborhood embedding, with each number representing a cluster. (b) Dot plots showing the average expression levels of selected canonical markers across identified cell clusters. Dot sizes indicate the proportion of cells expressing each marker within each cluster, while color represents relative expression intensity. (c) UMAP visualizations representing the single-cell transcriptomic profiles of each sample. Pathway enrichment analysis of Tumor_C1 (d) and Tumor_C2 (e) clusters.


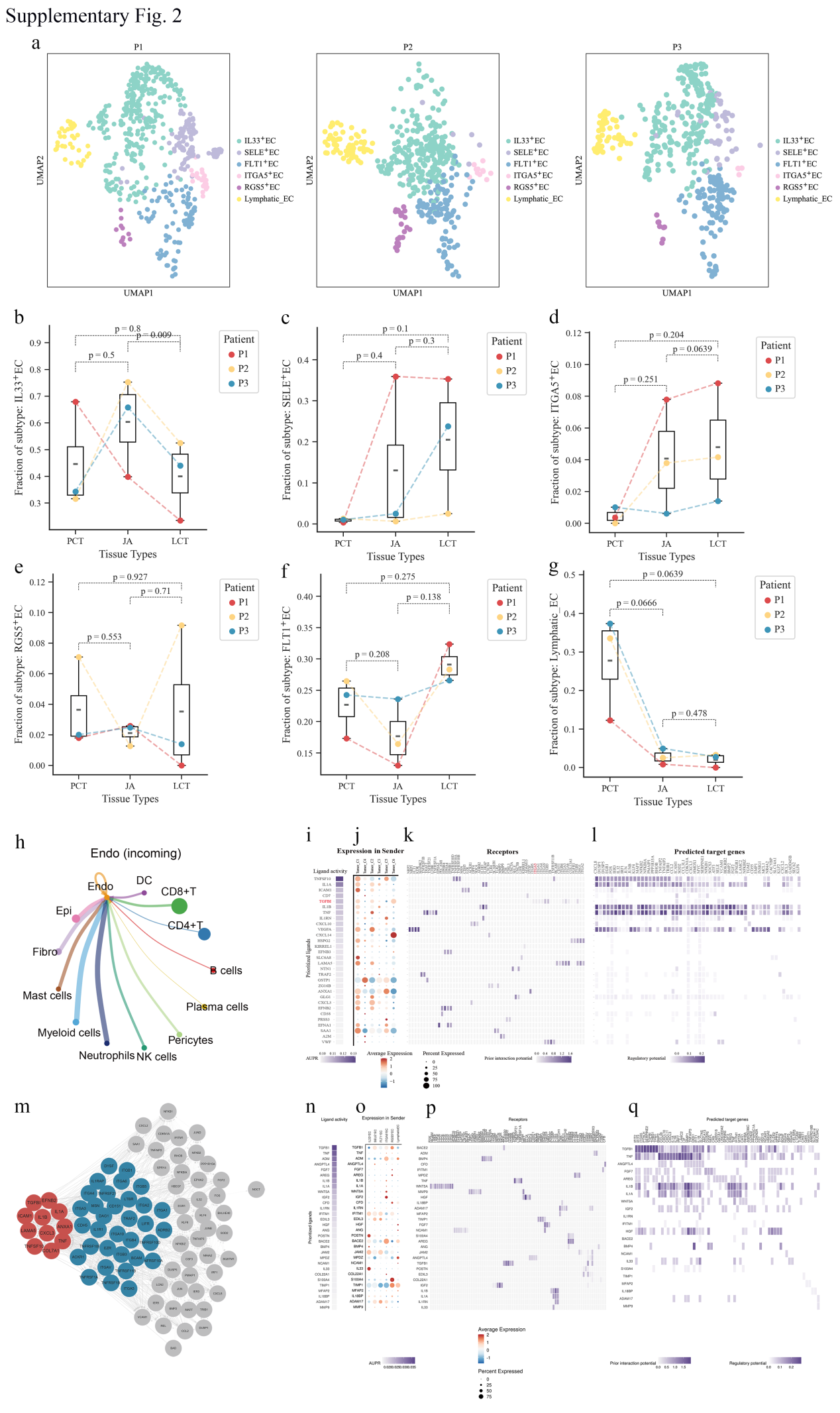


**Supplementary Fig. 2 Heterogeneous expression patterns of EC subpopulations across distinct tissue types, including PCT, JA, and LCT**(a) UMAP visualizations representing the EC profiles of each sample. Expression of IL33^+^ ECs (b), SELE^+^ ECs (c), ITGA5^+^ ECs (d), RGS5^+^ ECs (e), FLT1^+^ ECs (f), and Lymphatic ECs (g) in PCT, JA, and LCT of LSCC samples. Statistical significance was determined using the Wilcoxon rank-sum test. (h) Incoming interaction weights with endothelial cells as the receiver, other marjor cell types as the sender. Edge width encodes the CellChat-estimated interaction strength, and node size reflects cluster abundance. (i) Top-ranked ligands inferred to regulate ITGA5^+^ ECs by tumor cells according to NicheNet. (j) Dot plots showing the expression percentage (dot size) and intensity (dot intensity) of top-ranked ligands (i) in each tumor subtype. (k) Ligand**–**receptor pairs illustrating the interaction between Tumor cells and ITGA5^+^ ECs ordered by ligand activity (i). (l) Heatmap displaying the regulatory potential of top ligands (i) and the downstream target genes in ITGA5^+^ ECs. (m) Diagrams depicting the network of top ligands expressed by Tumor_C1 for receptor genes and target genes in ITGA5^+^ ECs. (n) Top ligands from the six endothelial subtypes (senders) predicted by NicheNet to act on Tumor_C2 cells (receiver), ranked by ligand activity. (o) Dot plots across the six endothelial subtypes (senders) showing, for each ligand in (n), the fraction of ligand-positive cells (dot size) and mean expression (color). (p) Predicted ligand–receptor pairs mediating endothelial to Tumor_C2 signaling, ordered by ligand activity in (n). (q) Heatmap of regulatory potential linking the ligands in (n) to downstream target genes within Tumor_C2 cells (receiver).


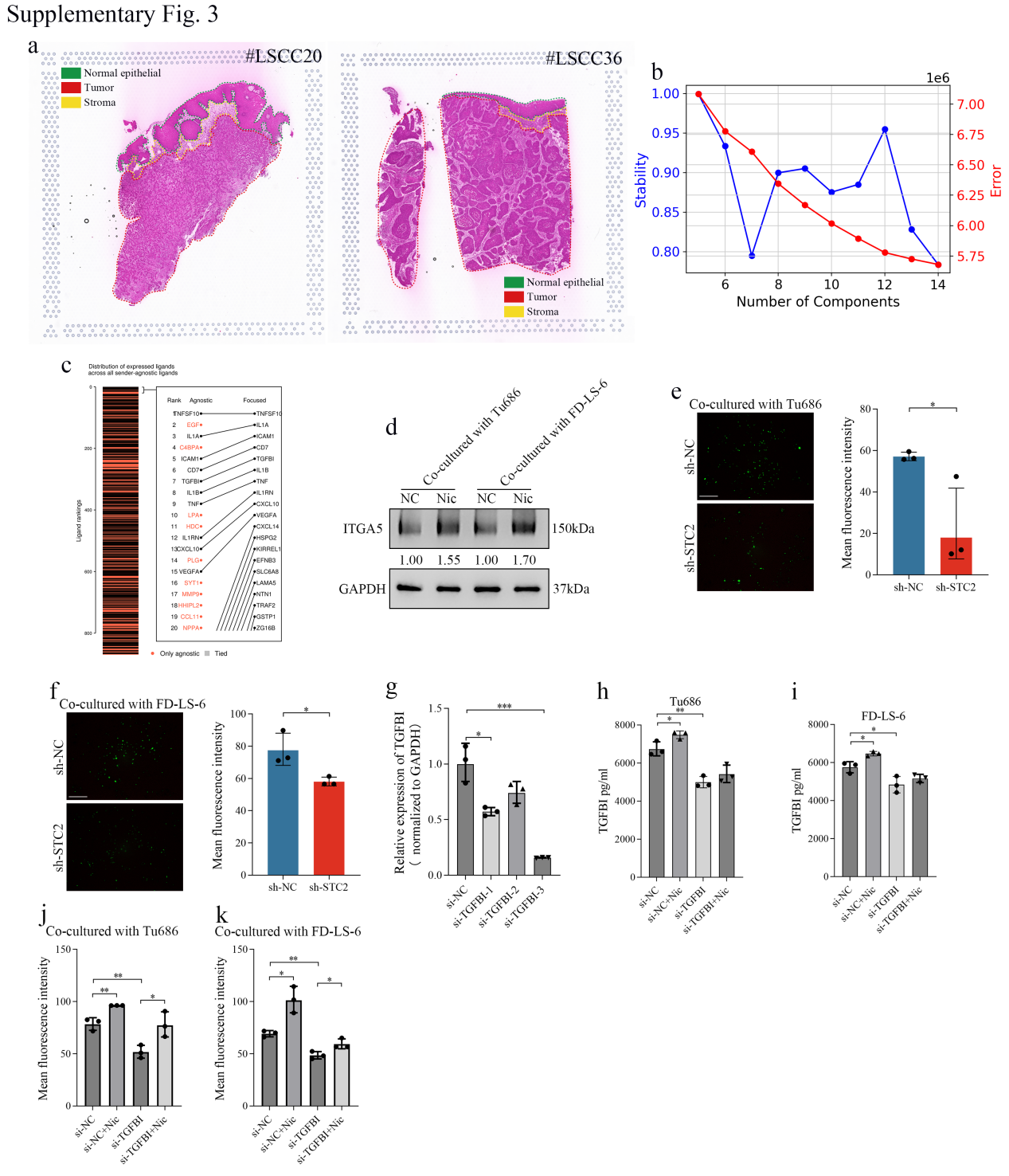


**Supplementary Fig. 3 Cellular communication features in the LSCC microenvironment**(a) H&E-stained tissue sections from LSCC individuals #20 (smoker) and #36 (non-smoker) with overlaid Visium spatial transcriptomics spots. Tumor regions, stroma, and adjacent normal epithelium are outlined. (b) Selection of the optimal number of components (k) for consensus non-negative matrix factorization (cNMF), showing the stability (blue) and error (red) of the cNMF model as a function of the number of components. (c) Concordance between expressed ligands (right) and all sender-agnostic ligands (left) across cell populations. (d) ITGA5 expression in ECs exposed to nicotine-conditioned medium compared with controls. GAPDH served as a loading control. Mean fluorescence intensity of ECs co-cultured with supernatant of Tu686 (e) and FD-LS-6 (f) cells infected with sh-NC or sh-STC2 lentiviruses. Scale bar: 500 μm. Data are presented as mean ± SD (n = 3). Statistical significance was determined using the unpaired t test, **p* < 0.05. (g) Confirmation of TGFBI mRNA expression in Tu686 cells infected with si‐NC, si‐TGFBI-1, si‐TGFBI-2, and si‐TGFBI-3. GAPDH served as a loading control. Data are presented as mean ± SD (n = 3). Statistical significance was determined using the unpaired t test, **p* < 0.05; ****p* < 0.001. Alteration of TGFBI protein levels in the supernatant of Tu686 (h) and FD-LS-6 (i) cells in si-NC, si-NC+Nic, si-TGFBI and si-TGFBI+Nic groups. Data are presented as mean ± SD (n = 3). Statistical significance was determined using the unpaired t test, **p* < 0.05; ***p* < 0.01. Mean fluorescence intensity of ECs co-cultured with supernatant of Tu686 (j) and FD-LS-6 (k) cells in si-NC, si-NC+Nic, si-TGFBI and si-TGFBI+Nic groups. Scale bar: 500 μm. Data are presented as mean ± SD (n = 3). Statistical significance was determined using the unpaired t test, **p* < 0.05; ***p* < 0.01.


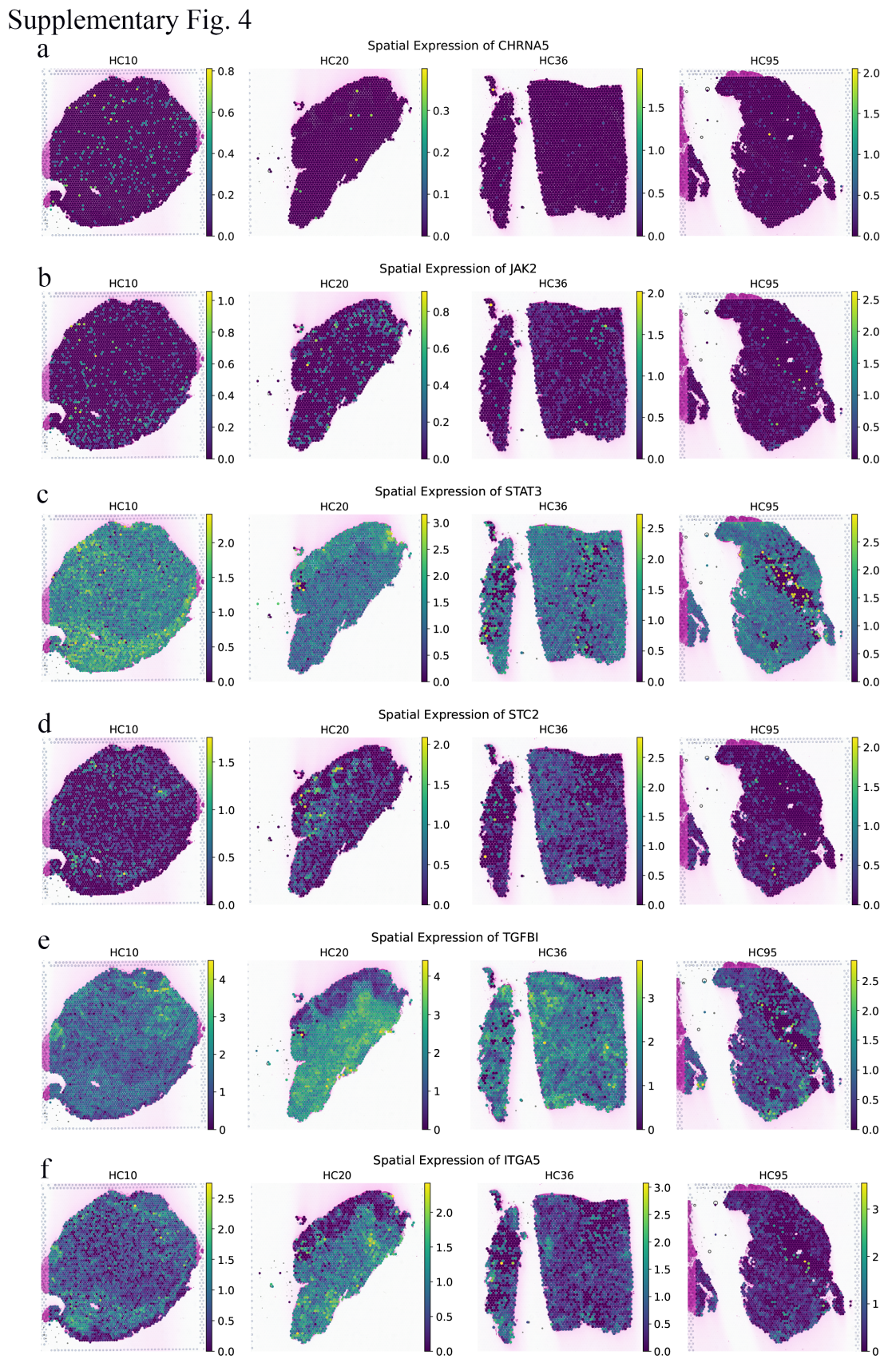


**Supplementary Fig. 4 Heterogeneity in spatial cell distribution and gene expression patterns within LSCC**The expression and spatial distribution of CHRNA5 (a), JAK2 (b), STAT3 (c), STC2 (d), TGFBI (e), and ITGA5 (f) in spatial transcriptome analysis specimens.


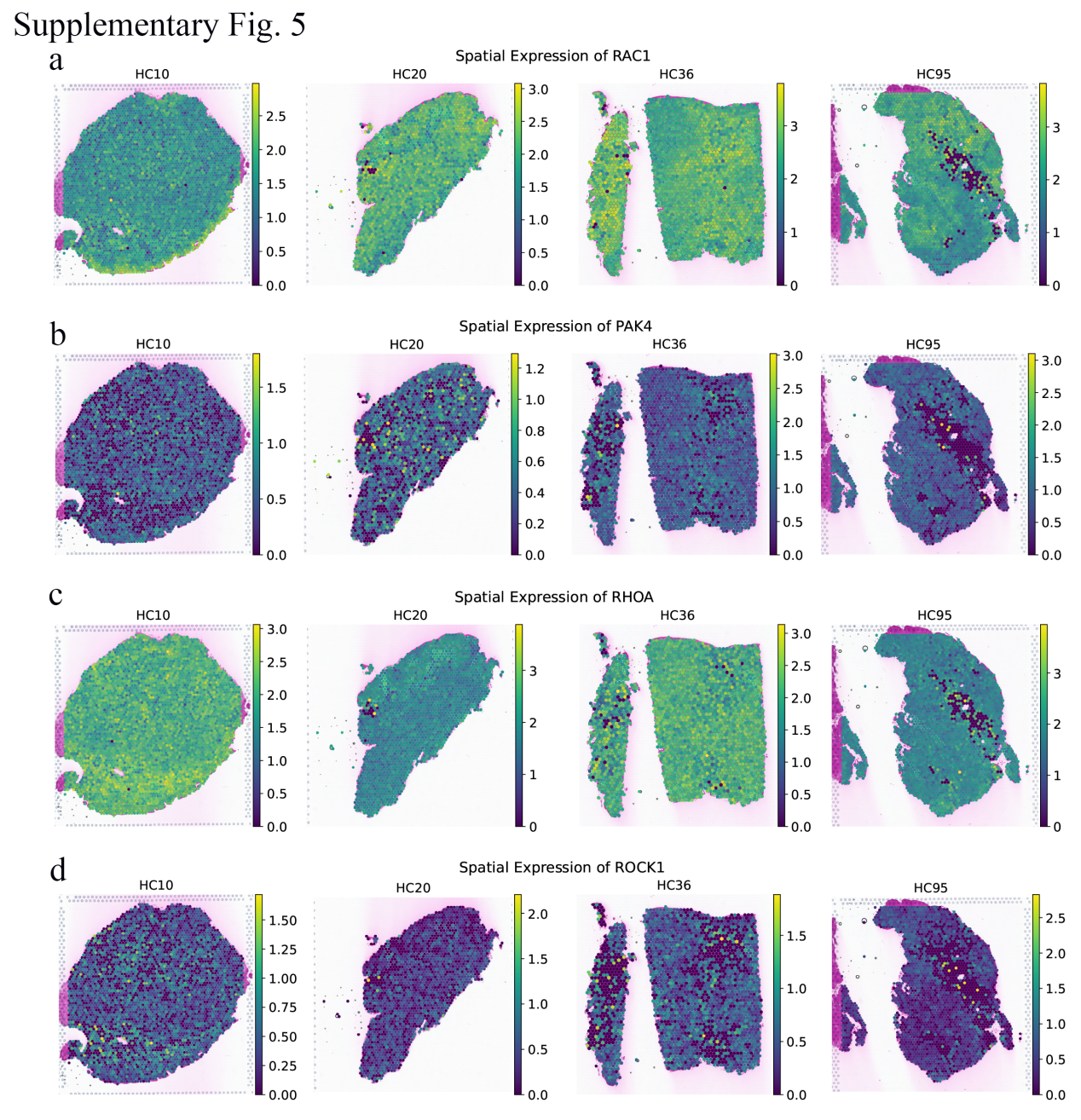


**Supplementary Fig. 5 Heterogeneity in spatial cell distribution and gene expression patterns within LSCC**The expression and spatial distribution of small GTPase signaling molecules RAC1 (a), PAK4 (b), RHOA (c), and ROCK1 (d) in spatial transcriptome analysis specimens.

**
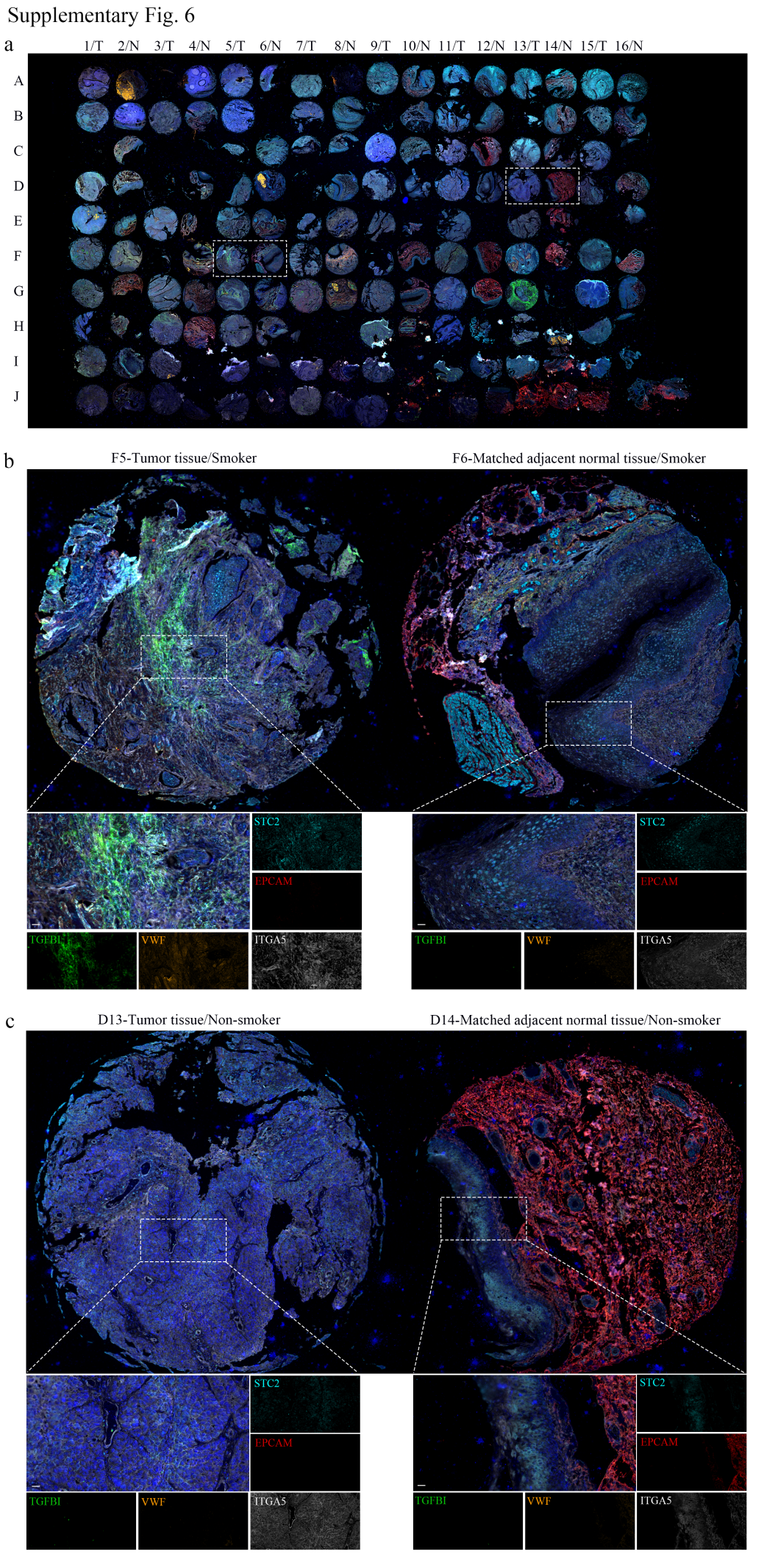
**

**Supplementary Fig. 6 Multiplex immunofluorescence validation of STC2–TGFBI–ITGA5 axis in LSCC**

(a) Representative staining images from a tissue microarray containing 61 pairs of LSCC tumors and matched adjacent normal tissues (detailed clinical information in Supplementary Table 9). Representative multiplex immunofluorescence staining results of smoking (b) and non-smoking (c) LSCC samples (tumor and matched adjacent normal tissues) stained for STC2 (cyan), EPCAM (red), ITGA5 (white), VWF (orange), TGFBI (green), and DAPI (blue). Scale bar: 50 μm.

**
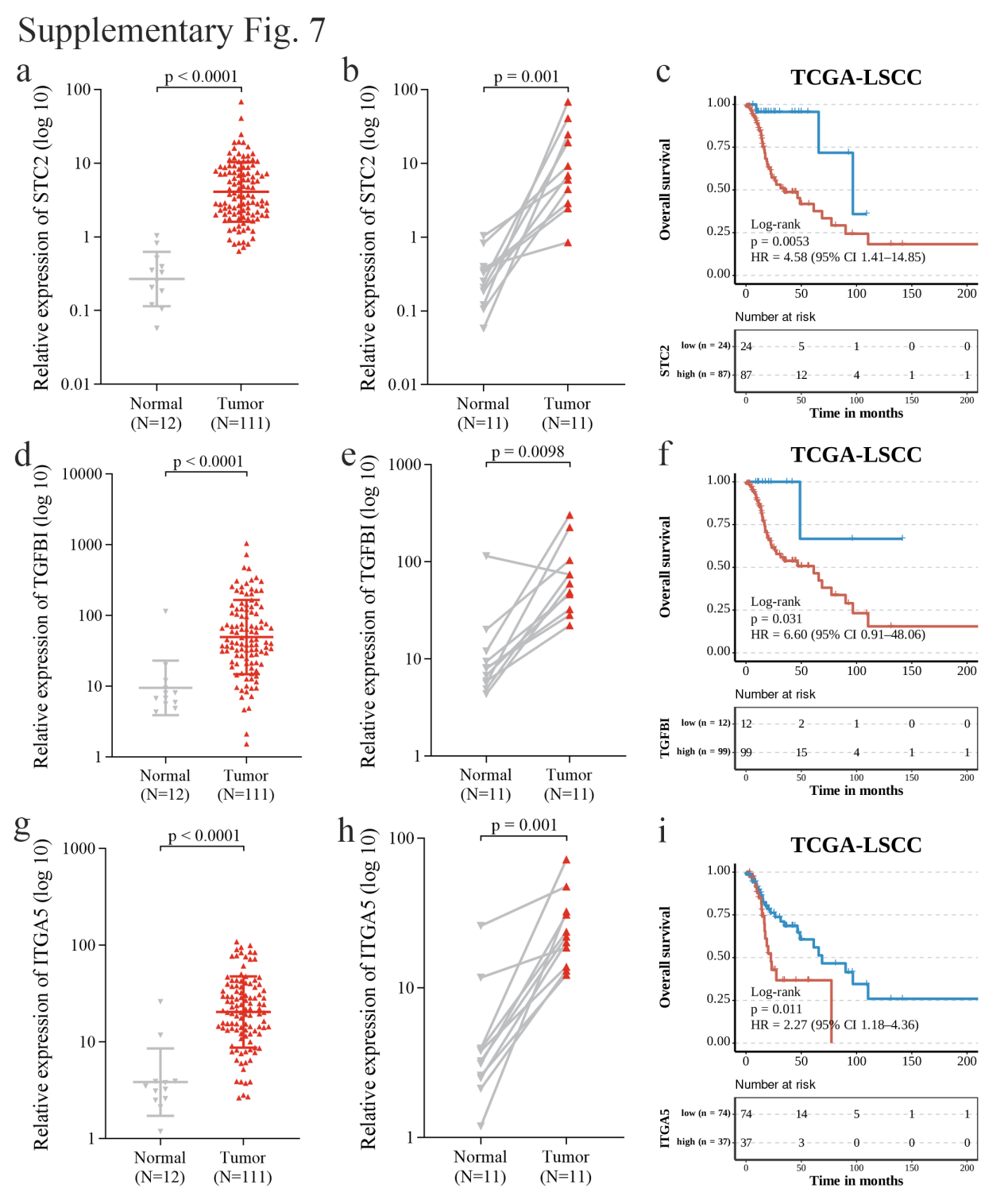
**

**Supplementary Fig. 7 Expression of key genes in the TCGA-LSCC cohort**

Expression of STC2 (a), TGFBI (d), ITGA5 (g) in LSCC tumor tissues compared with adjacent normal tissues (Mann-Whitney U test). Expression of STC2 (b), TGFBI (e), ITGA5 (h) in paired LSCC tumor tissues relative to matched adjacent normal tissues (n = 11, two-sided Wilcoxon signed-rank test). Kaplan-Meier curves of patients with LSCC stratified by STC2 (c), TGFBI (f), and ITGA5 (i) expression. The optimal cutoff threshold was used for stratification, *p*-values were calculated using the Log-rank test.


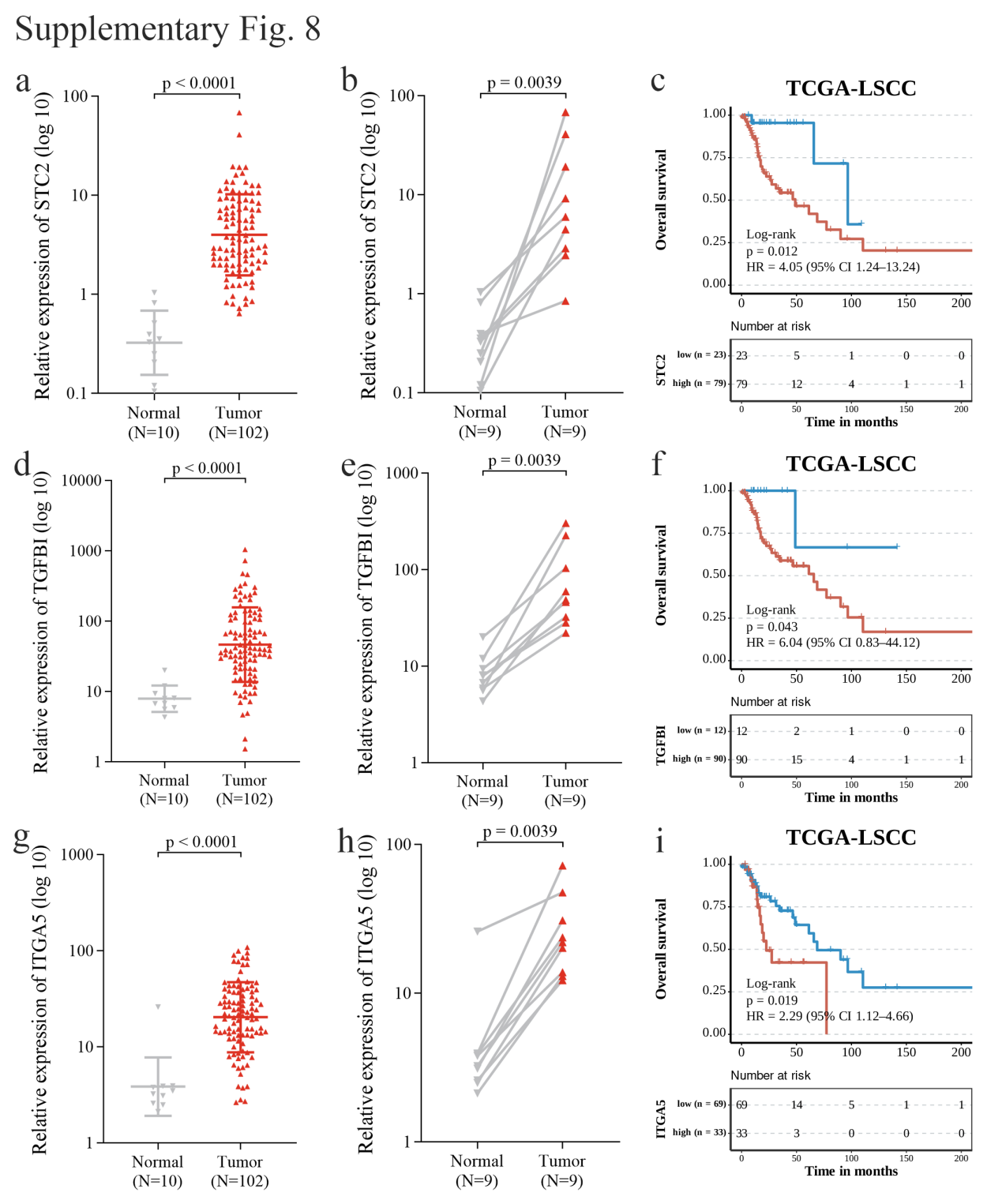


**Supplementary Fig. 8 Expression of key genes in the smoking-associated TCGA-LSCC cohort**

Expression of STC2 (a), TGFBI (d), ITGA5 (g) in smoking LSCC tumor tissues compared with adjacent normal tissues (Mann-Whitney U test). Expression of STC2 (b), TGFBI (e), ITGA5 (h) in paired smoking LSCC tumor tissues relative to matched adjacent normal tissues (n = 9, two-sided Wilcoxon signed-rank test). Kaplan-Meier curves of patients with smoking LSCC stratified by STC2 (c), TGFBI (f), and ITGA5 (i) expression. The optimal cutoff threshold was used for stratification, *p*-values were calculated using the Log-rank test.
